# Supplementary figures and images for: Modeling the onset of symptoms of COVID-19: Effects of SARS-CoV-2 variant
Source: PLoS Comput Biol. 2021 Dec 16;17(12):e1009629. doi: 10.1371/journal.pcbi.1009629 (PMC8675677; doi:10.1371/journal.pcbi.1009629)

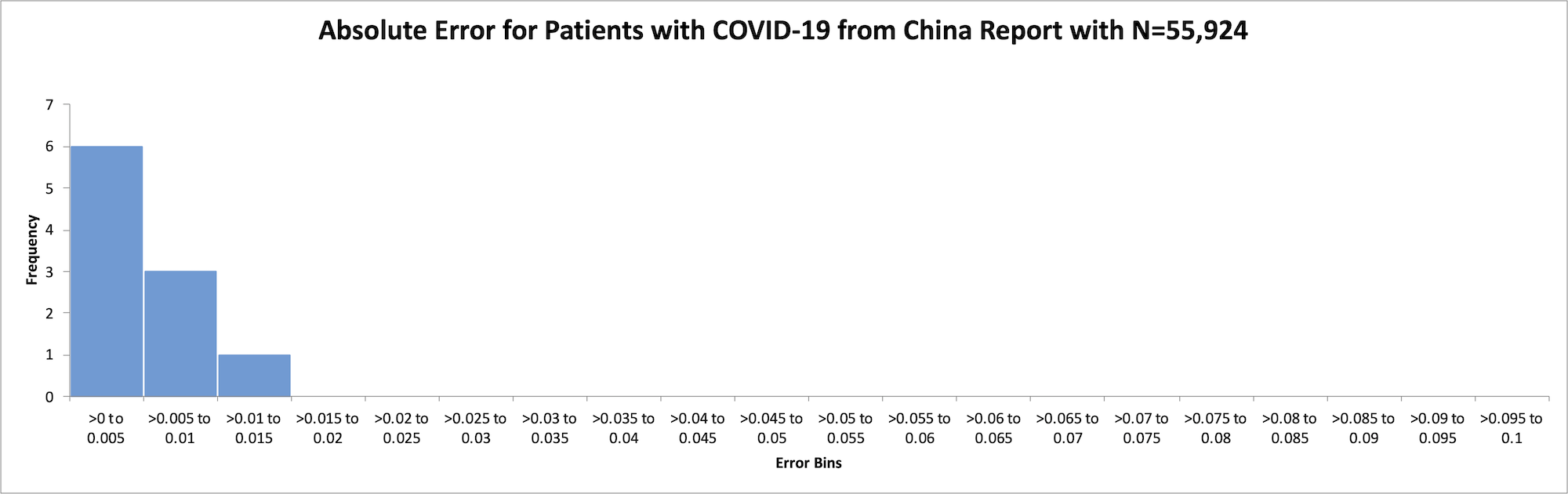

Supplement: S1 Fig — The maximum error was determined from this distribution of absolute error and was used as a conservative measure of error to discern differences in transition probabilities of discernible symptom order. (TIF) [file pcbi.1009629.s006.tif]

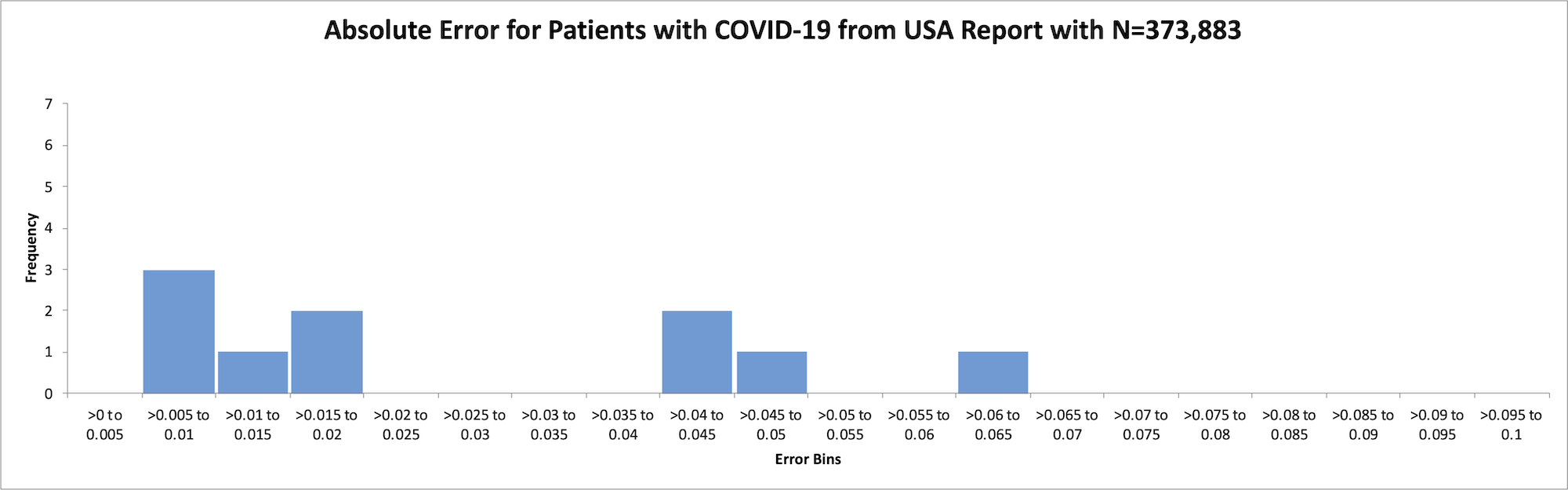

Supplement: S2 Fig — The maximum error was determined from this distribution of absolute error and was used as a conservative measure of error to discern differences in transition probabilities of discernible symptom order. (TIF) [file pcbi.1009629.s007.tif]

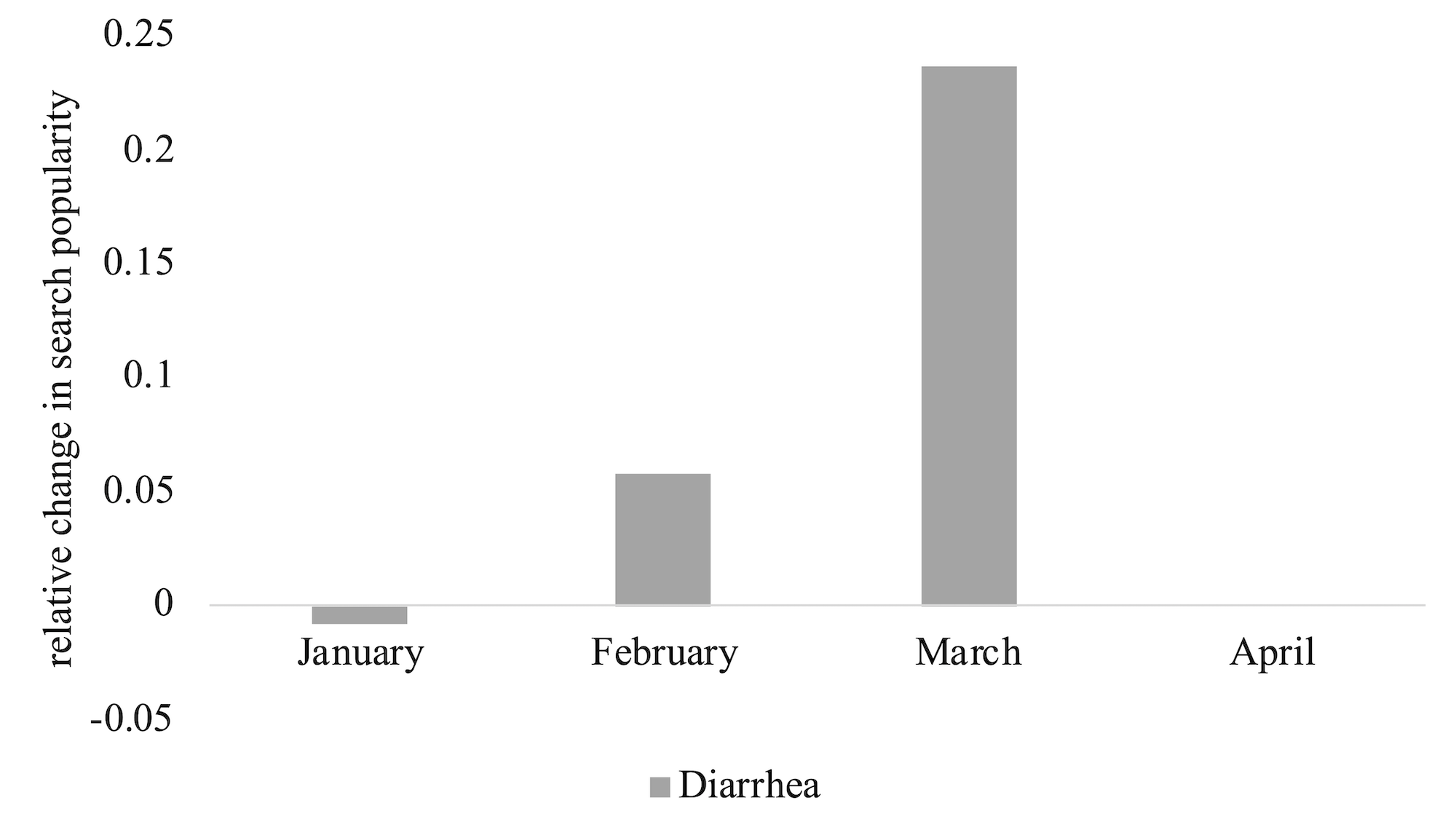

Supplement: S3 Fig — A bar graph displaying the relative change, compared to the previous year, of search popularity of the term, “diarrhea”, in the months, January, February, March, and April 2020 calculated from Google Trends [21]. (TIF) [file pcbi.1009629.s008.tif]

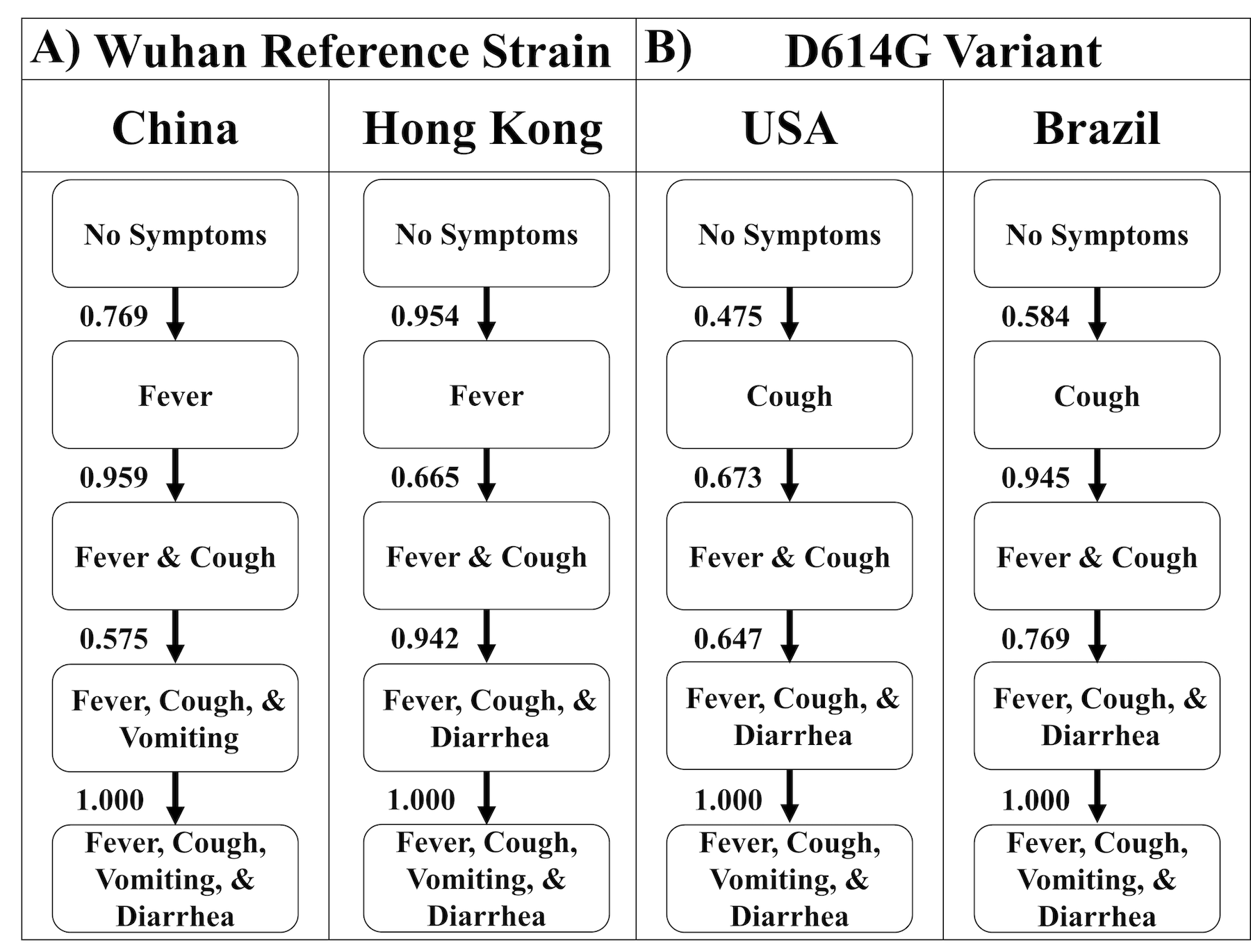

Supplement: S4 Fig — A) The most likely paths of discernible symptom order using datasets from China and Hong Kong, which are both characterized by the Wuhan reference strain, are displayed. B) The most likely paths of discernible symptom order using datasets from the USA and Brazil, which are both characterized by the D614G variant, are displayed. (TIF) [file pcbi.1009629.s009.tif]

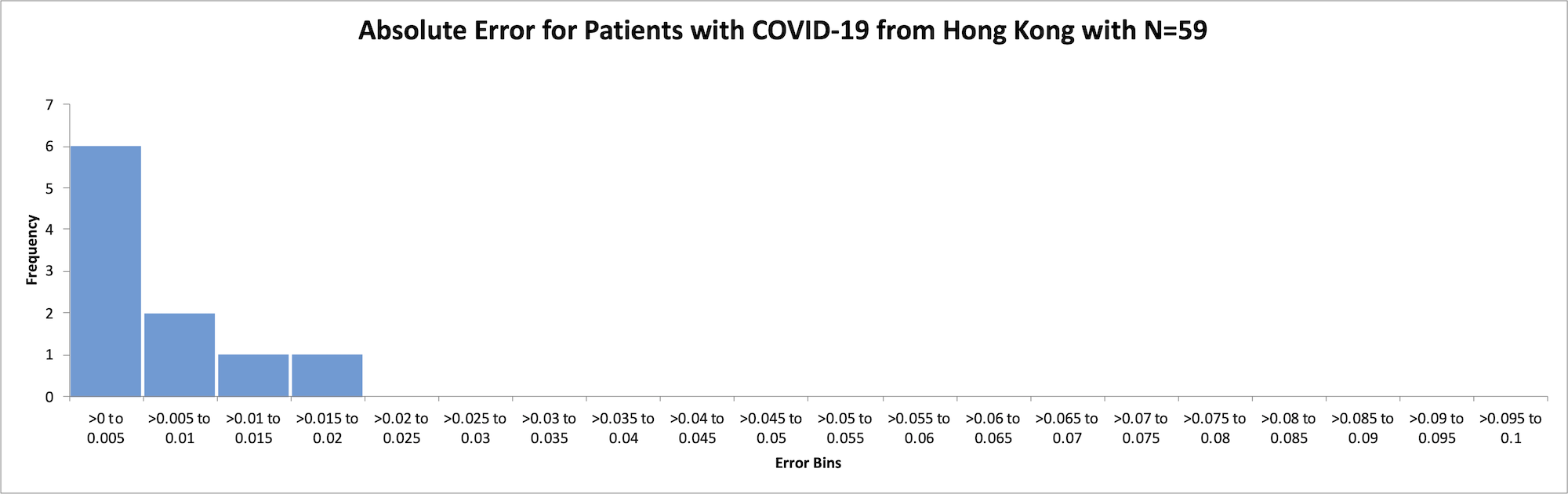

Supplement: S5 Fig — The maximum error was determined from this distribution of absolute error and was used as a conservative measure of error to discern differences in transition probabilities of discernible symptom order. (TIF) [file pcbi.1009629.s010.tif]

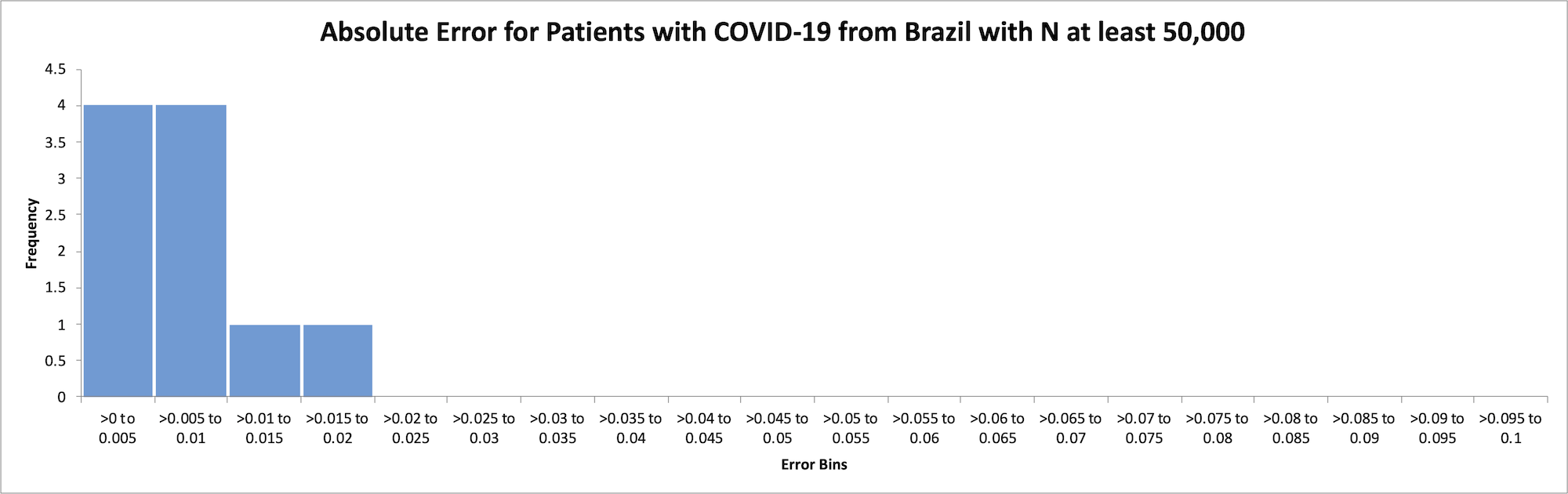

Supplement: S6 Fig — The maximum error was determined from this distribution of absolute error and was used as a conservative measure of error to discern differences in transition probabilities of discernible symptom order. (TIF) [file pcbi.1009629.s011.tif]

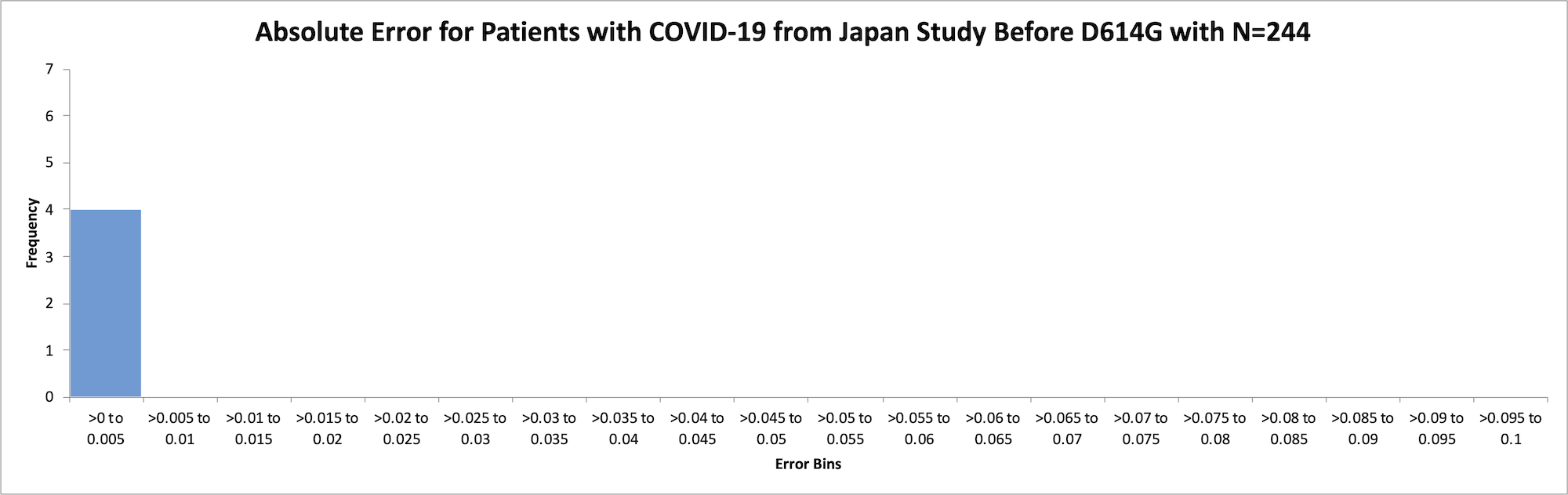

Supplement: S7 Fig — The maximum error was determined from this distribution of absolute error and was used as a conservative measure of error to discern differences in transition probabilities of discernible symptom order. (TIF) [file pcbi.1009629.s012.tif]

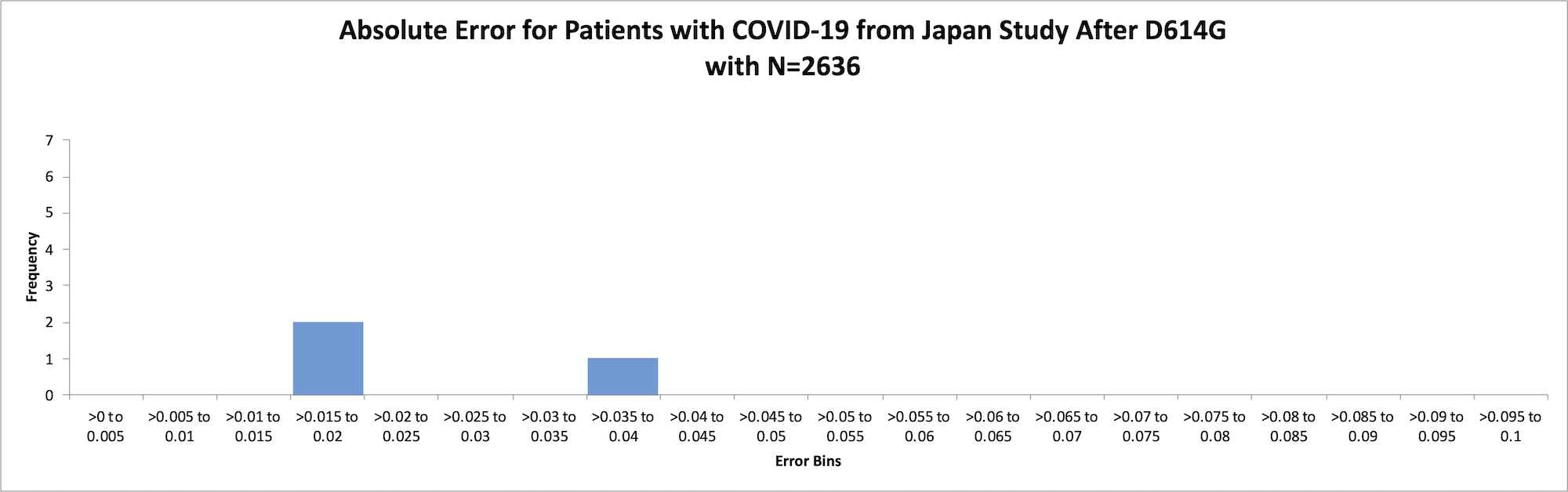

Supplement: S8 Fig — The maximum error was determined from this distribution of absolute error and was used as a conservative measure of error to discern differences in transition probabilities of discernible symptom order. (TIF) [file pcbi.1009629.s013.tif]

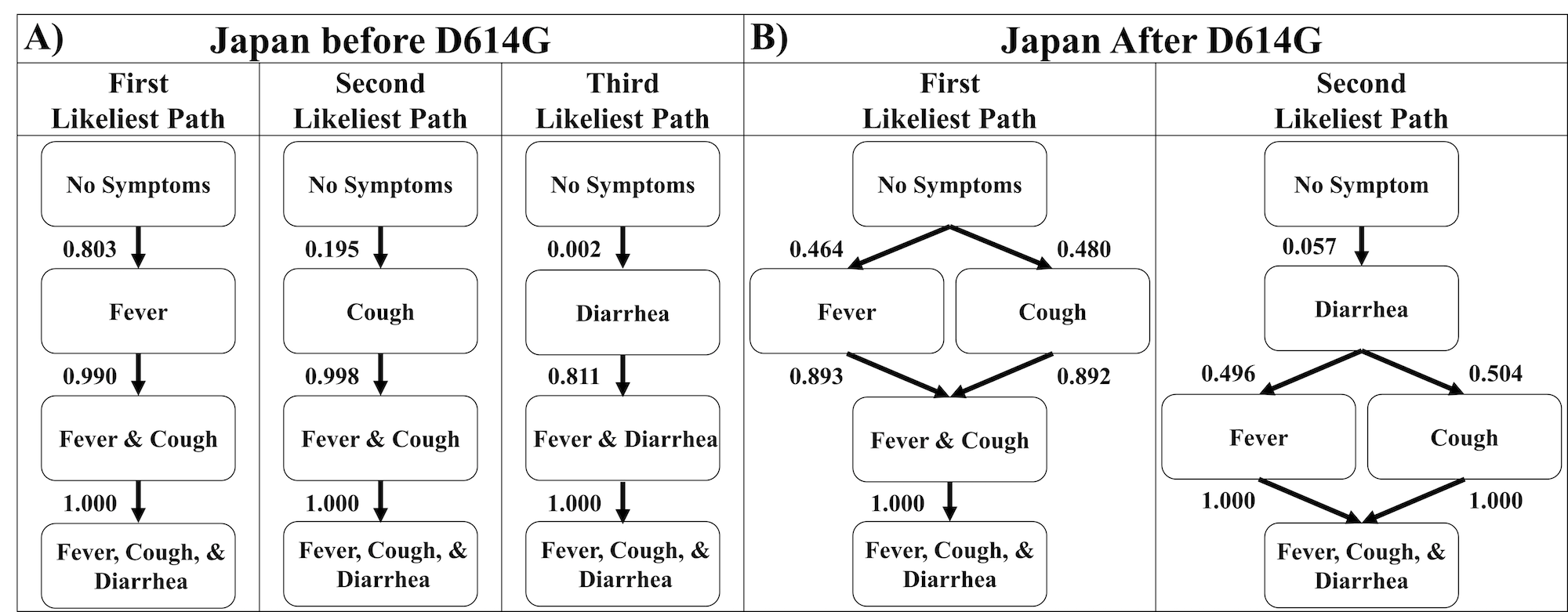

Supplement: S9 Fig — A) The first, second, and third most likely orders of discernible symptoms, with transition probabilities between symptoms of 244 COVID-19 patients in the Osaka Prefecture before the D614G mutation became prominent. B) The first, second, and third most likely orders of discernible symptoms, with transition probabilities between symptoms of 2,636 individuals with COVID-19 from Japan after the D614G mutation became prominent. (TIF) [file pcbi.1009629.s014.tif]

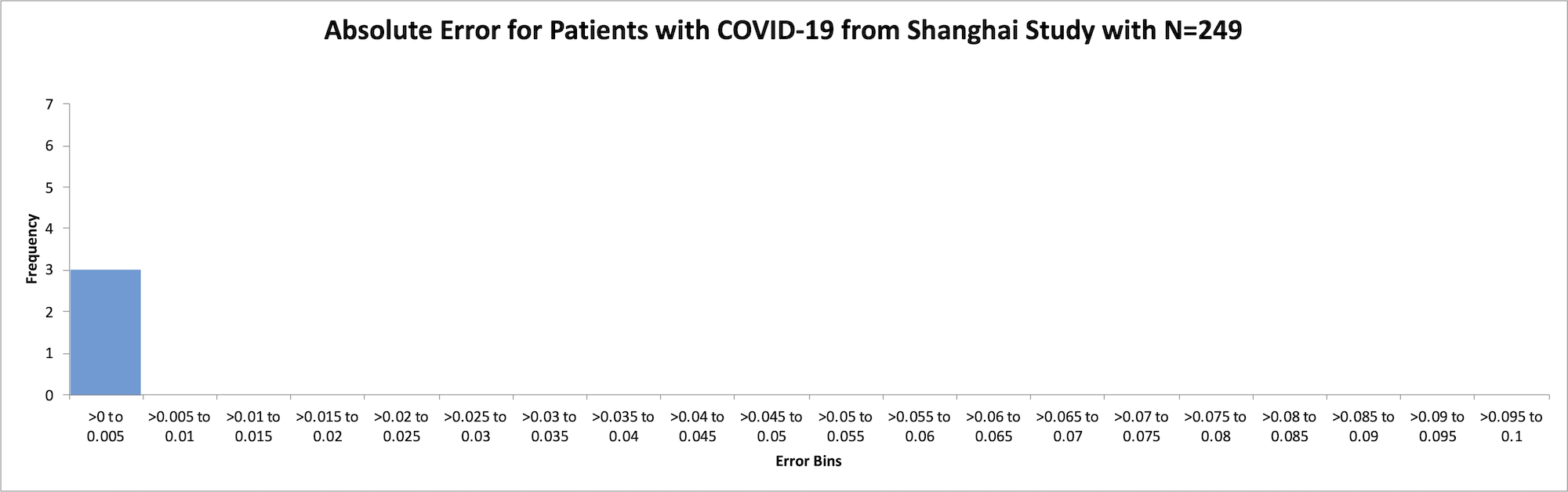

Supplement: S10 Fig — The maximum error was determined from this distribution of absolute error and was used as a conservative measure of error to discern differences in transition probabilities of discernible symptom order. (TIF) [file pcbi.1009629.s015.tif]

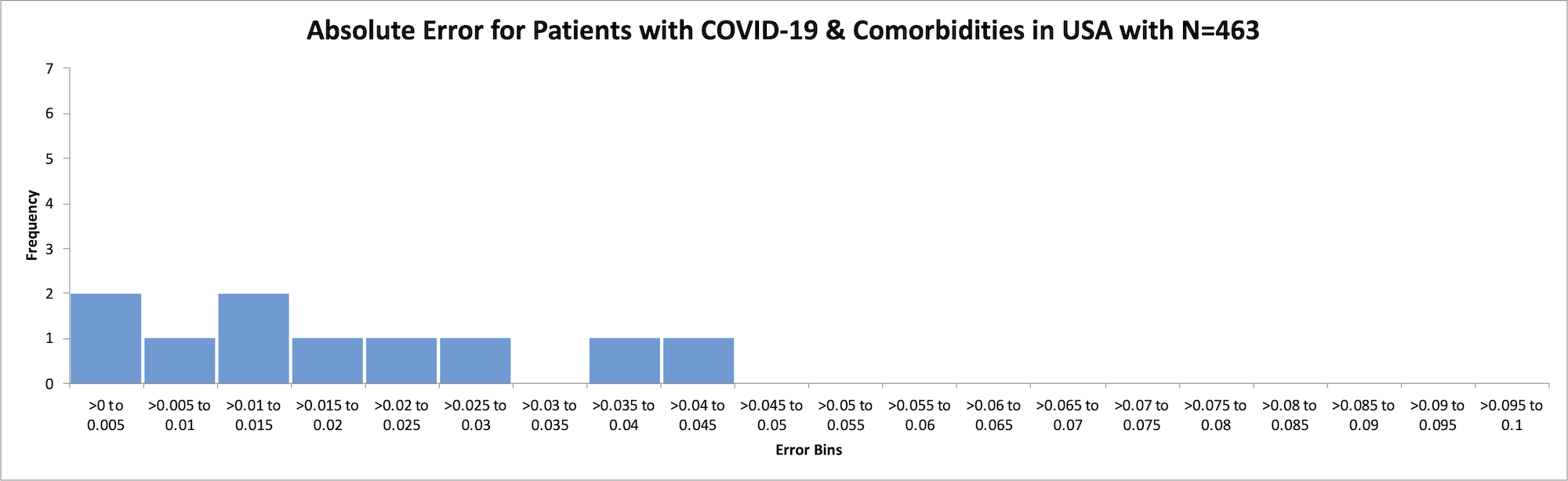

Supplement: S11 Fig — The maximum error was determined from this distribution of absolute error and was used as a conservative measure of error to discern differences in transition probabilities of discernible symptom order. (TIF) [file pcbi.1009629.s016.tif]

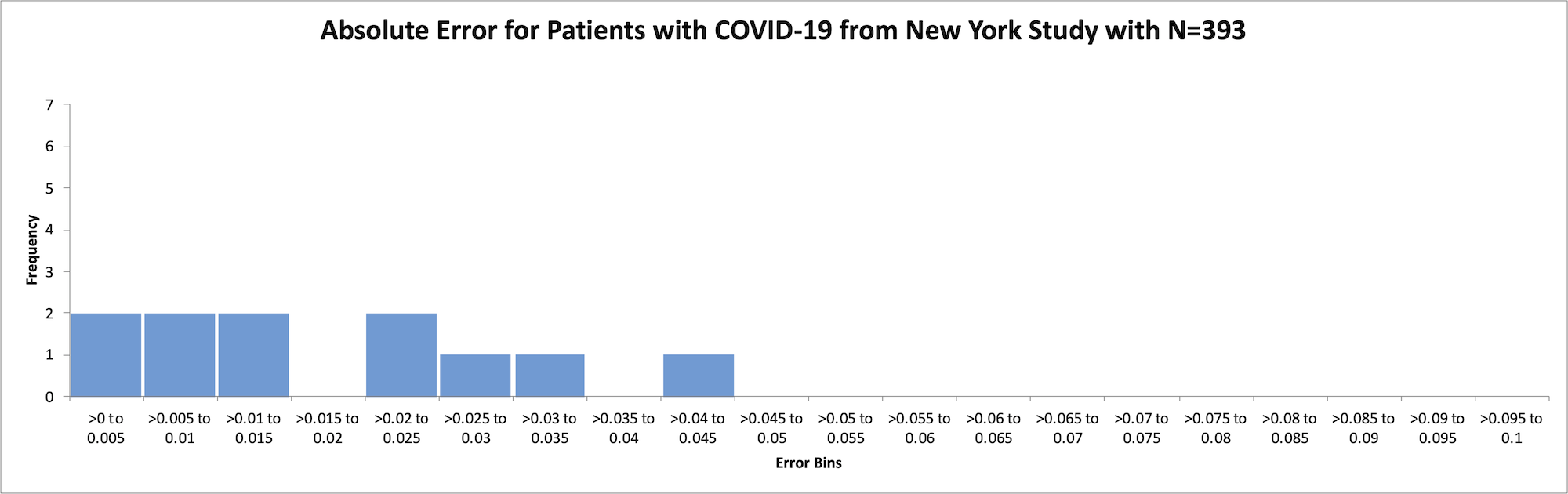

Supplement: S12 Fig — The maximum error was determined from this distribution of absolute error and was used as a conservative measure of error to discern differences in transition probabilities of discernible symptom order. (TIF) [file pcbi.1009629.s017.tif]

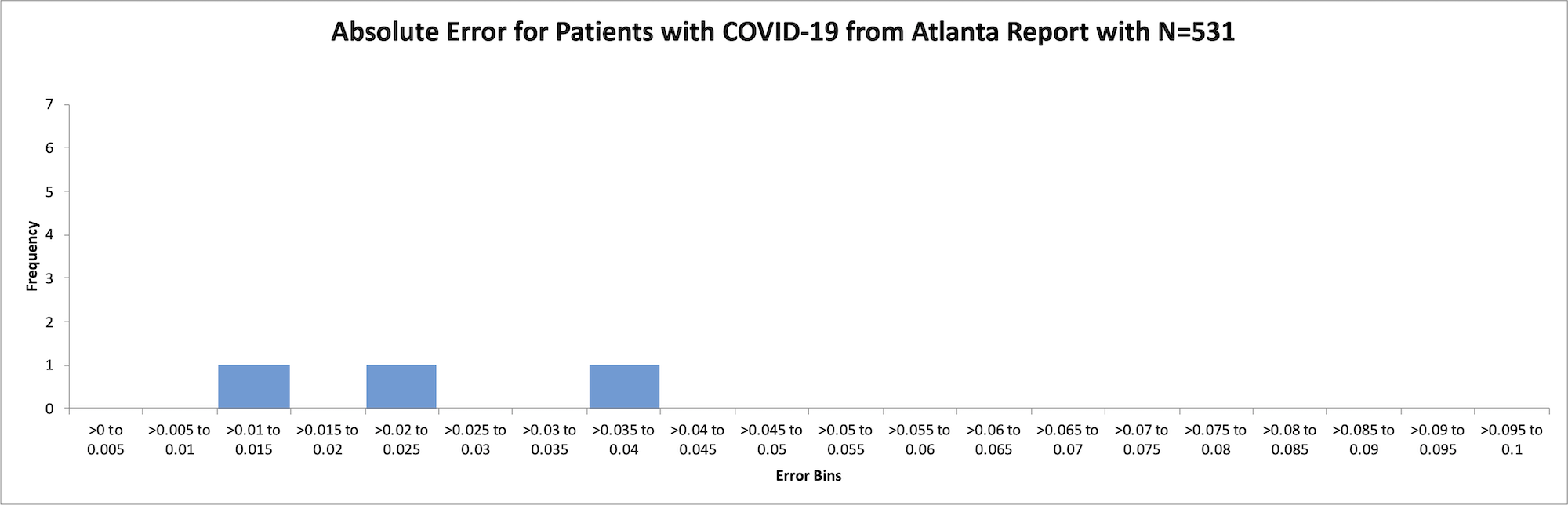

Supplement: S13 Fig — The maximum error was determined from this distribution of absolute error and was used as a conservative measure of error to discern differences in transition probabilities of discernible symptom order. (TIF) [file pcbi.1009629.s018.tif]

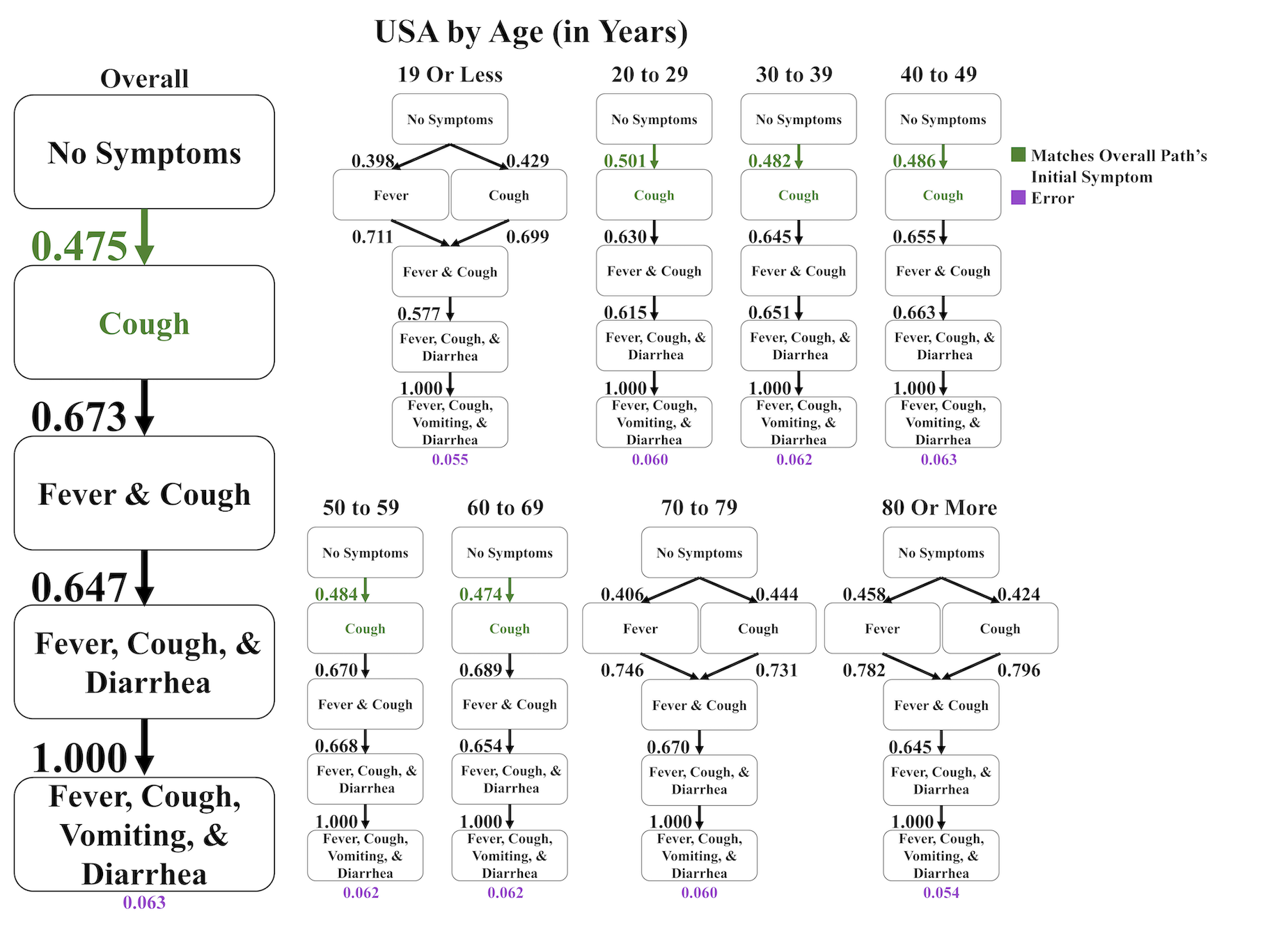

Supplement: S14 Fig — The most likely path of discernible symptom order for all patients in the USA dataset is shown on the left. All other columns are the most likely path of discernible symptom order by age groups from the overall set. The initial transition is written in green if it is consistent with the overall most likely path. The error of each implementation is written in purple. (TIF) [file pcbi.1009629.s019.tif]

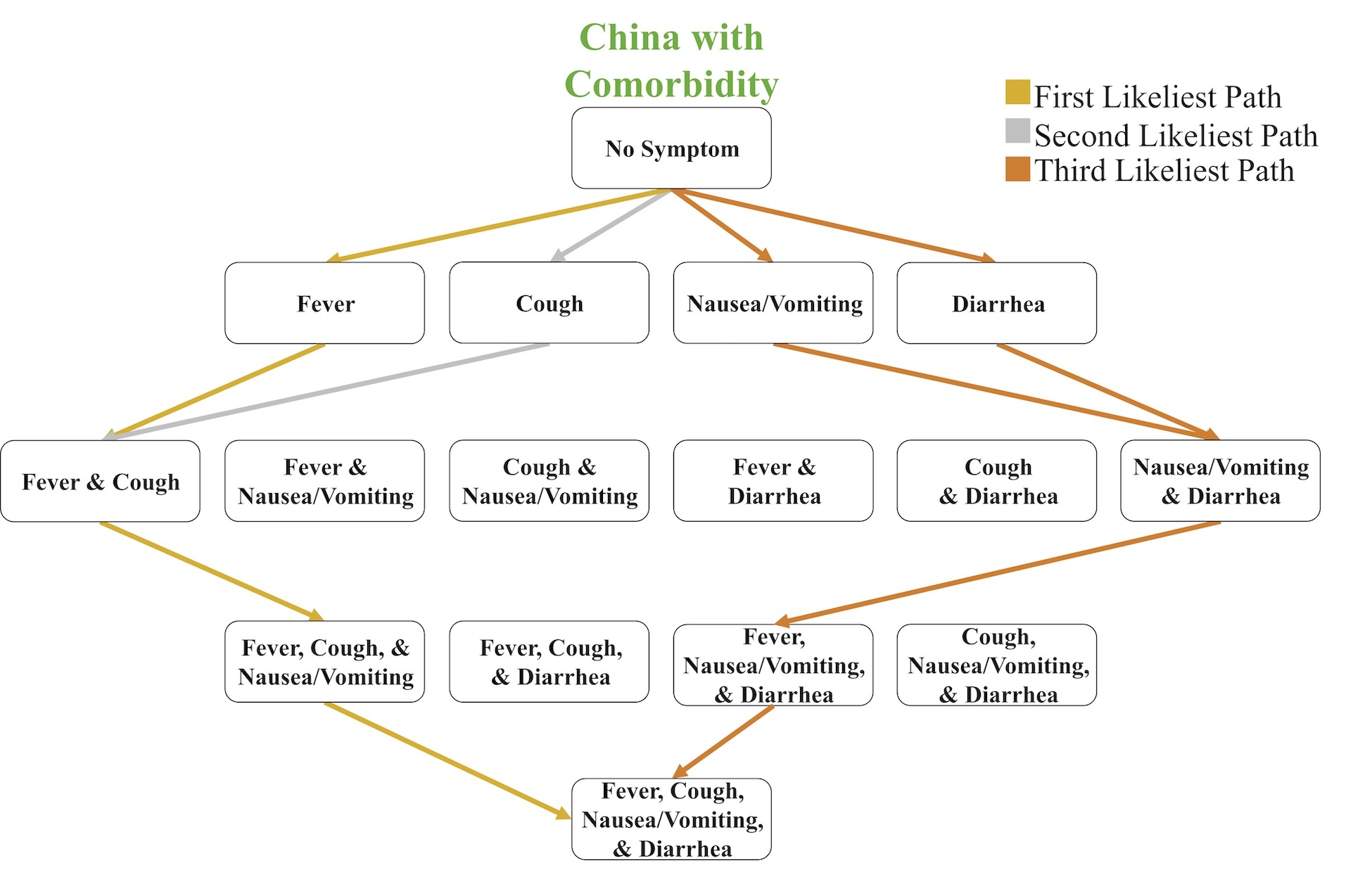

Supplement: S15 Fig — The first (gold), second (silver), and third (bronze) likeliest paths representing the order of discernible symptom onset of 399 individuals with COVID-19 and at least one comorbidity in China. In the case where the gold and silver lines converge, they both follow the same path. (TIF) [file pcbi.1009629.s020.tif]

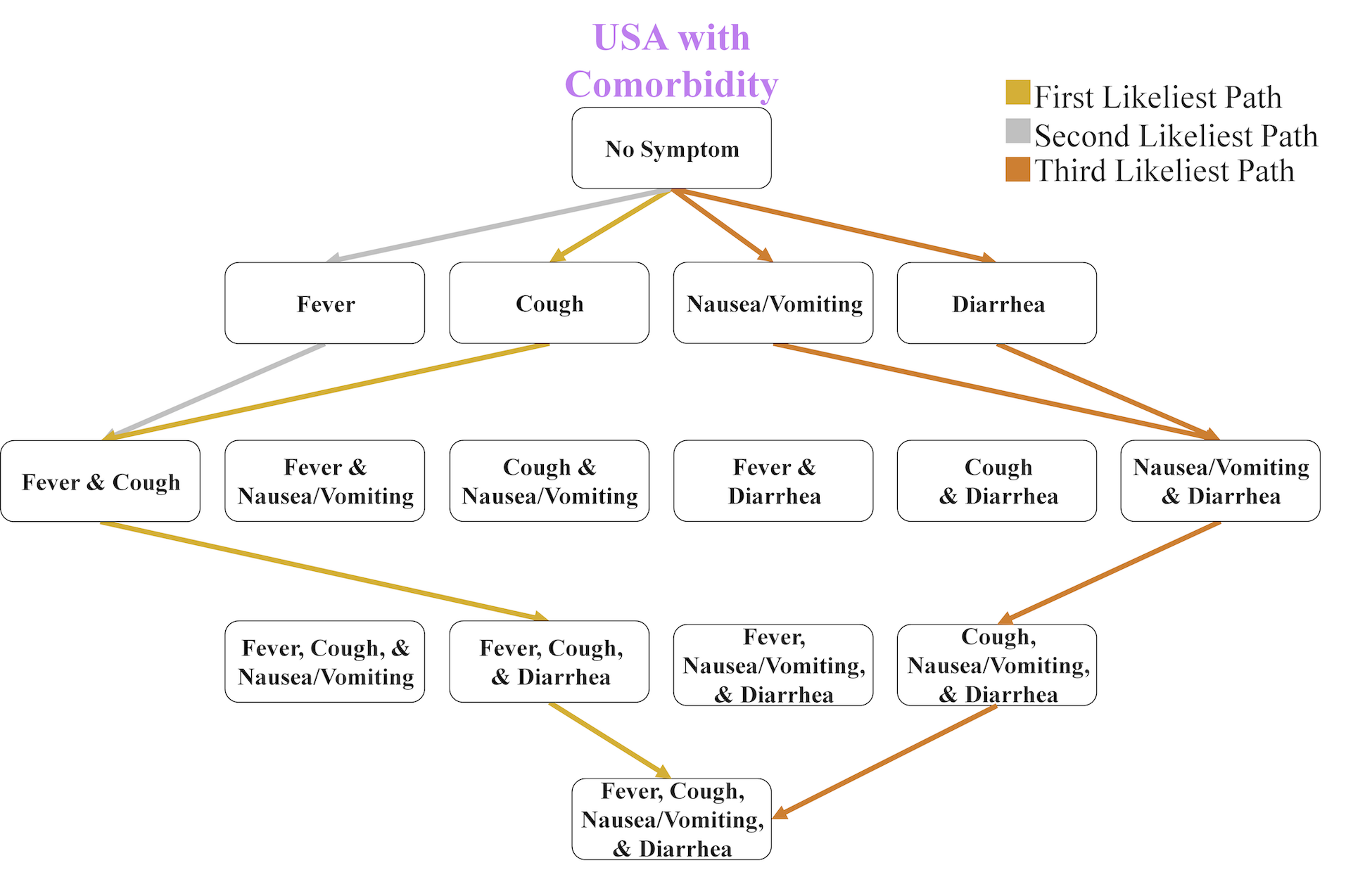

Supplement: S16 Fig — The first (gold), second (silver), and third (bronze) likeliest paths representing the order of discernible symptom onset of 463 individuals with COVID-19 and at least one comorbidity in Detroit, Michigan. In the case where the gold and silver lines converge, they both follow the same path. (TIF) [file pcbi.1009629.s021.tif]

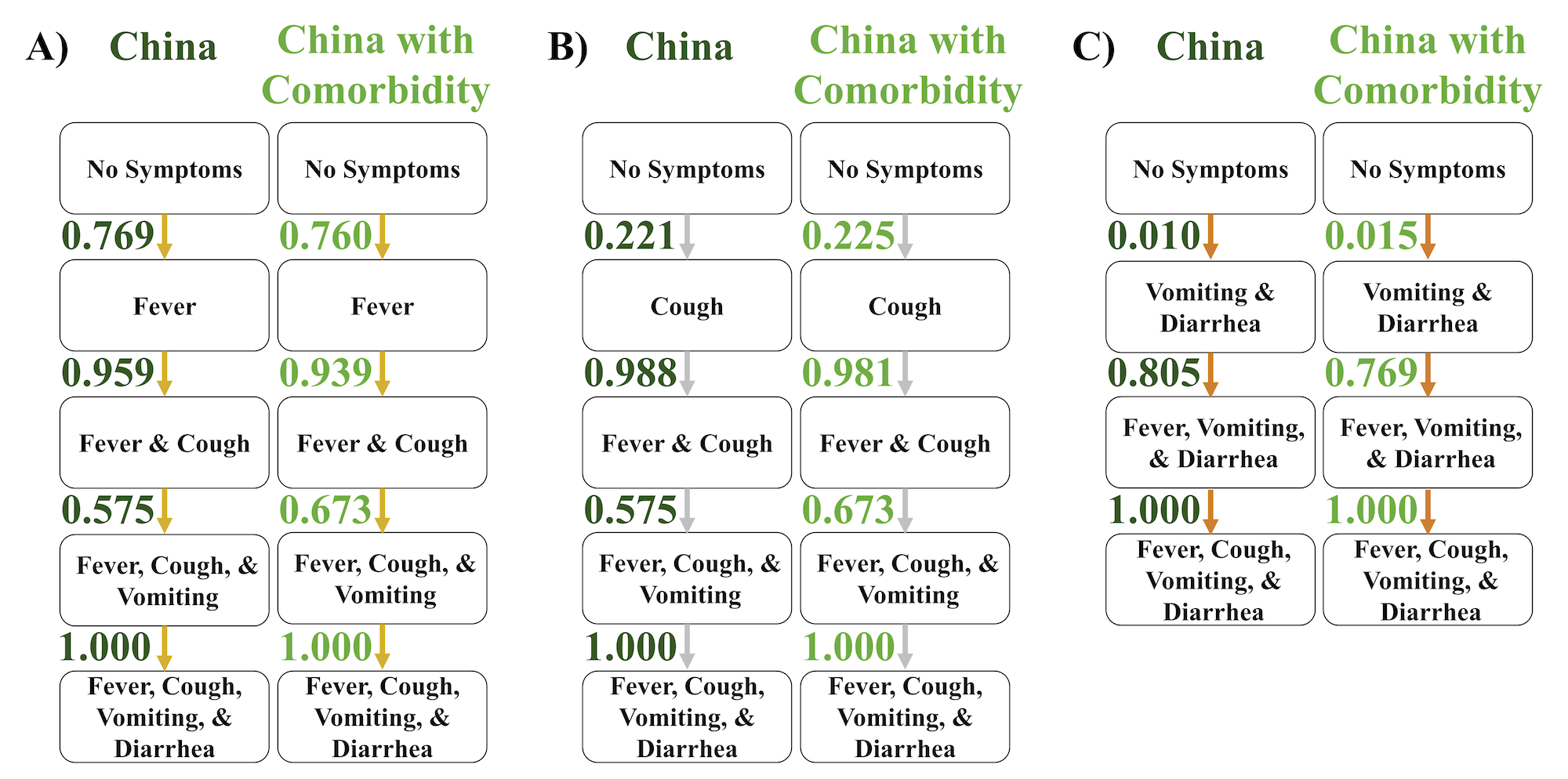

Supplement: S17 Fig — A) The first likeliest path (gold) of discernible symptom onset in China (green) and in China with at least one comorbidity (light green). B) The second likeliest path (silver) of discernible symptom onset in China (green) and in China with at least one comorbidity (light green). C) The third likeliest path (bronze) of discernible symptom onset in China (green) and in China with at least one comorbidity (light green). The error of the transition probabilities of the China dataset is 0.010, whereas the error of the transition probabilities of the China with comorbidities dataset is 0.013. (TIF) [file pcbi.1009629.s022.tif]

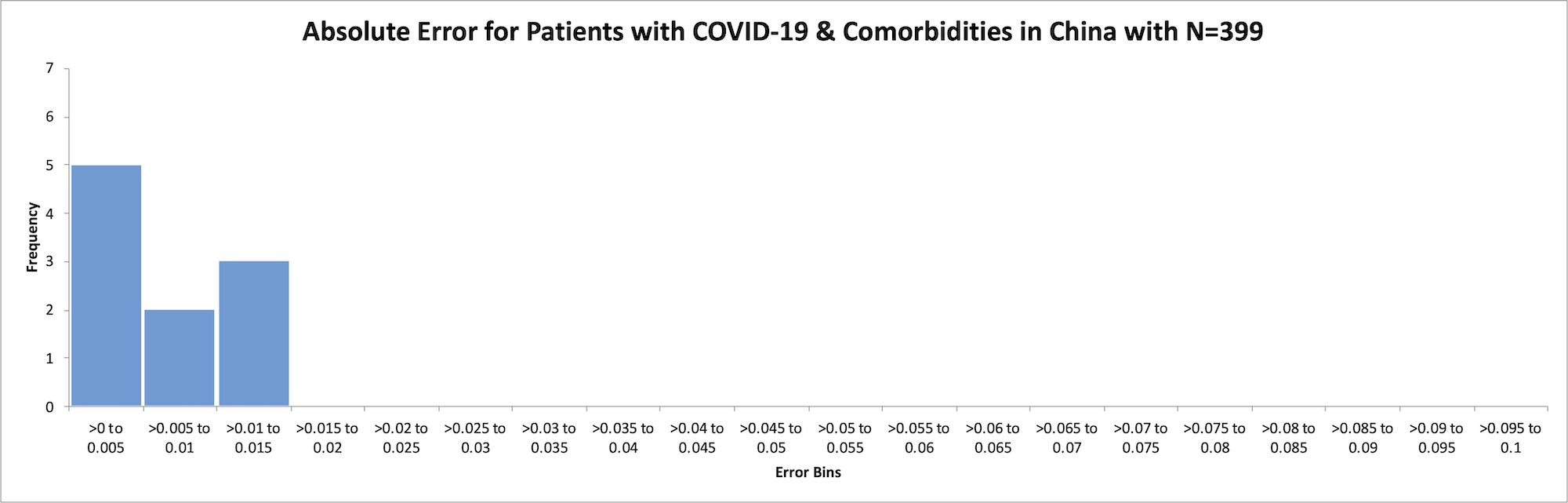

Supplement: S18 Fig — The maximum error was determined from this distribution of absolute error and was used as a conservative measure of error to discern differences in transition probabilities of discernible symptom order. (TIF) [file pcbi.1009629.s023.tif]

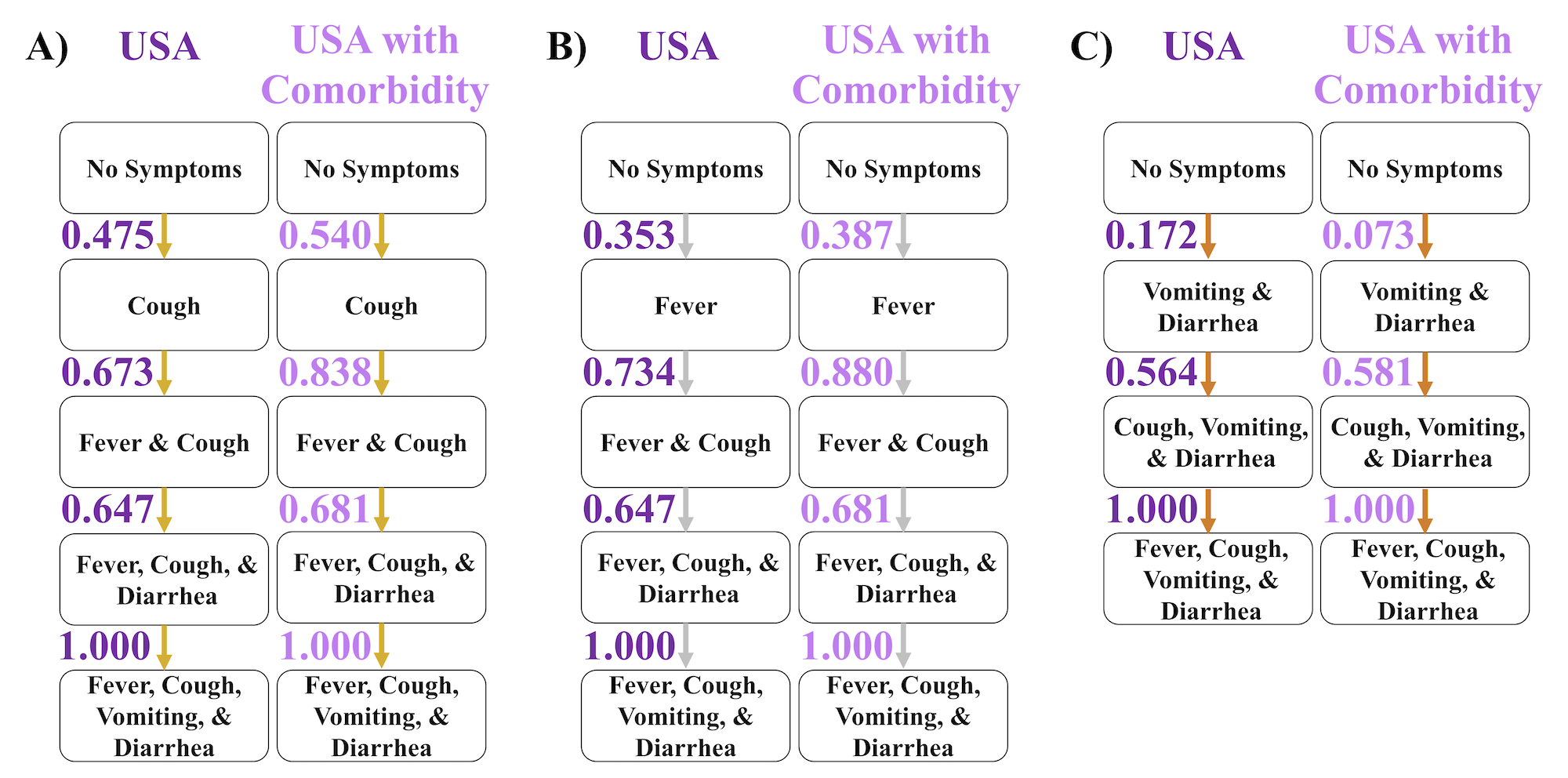

Supplement: S19 Fig — A) The first likeliest path (gold) of discernible symptom onset in USA (purple) and in Detroit with at least one comorbidity (light purple). B) The second likeliest path (silver) of discernible symptom onset in USA (purple) and in Detroit with at least one comorbidity (light purple). C) The third likeliest path (bronze) of discernible symptom onset in USA (purple) and in Detroit with at least one comorbidity (light purple). The error of the transition probabilities of the USA dataset is 0.063, whereas the error of the transition probabilities of the USA with comorbidities dataset is 0.044. (TIF) [file pcbi.1009629.s024.tif]

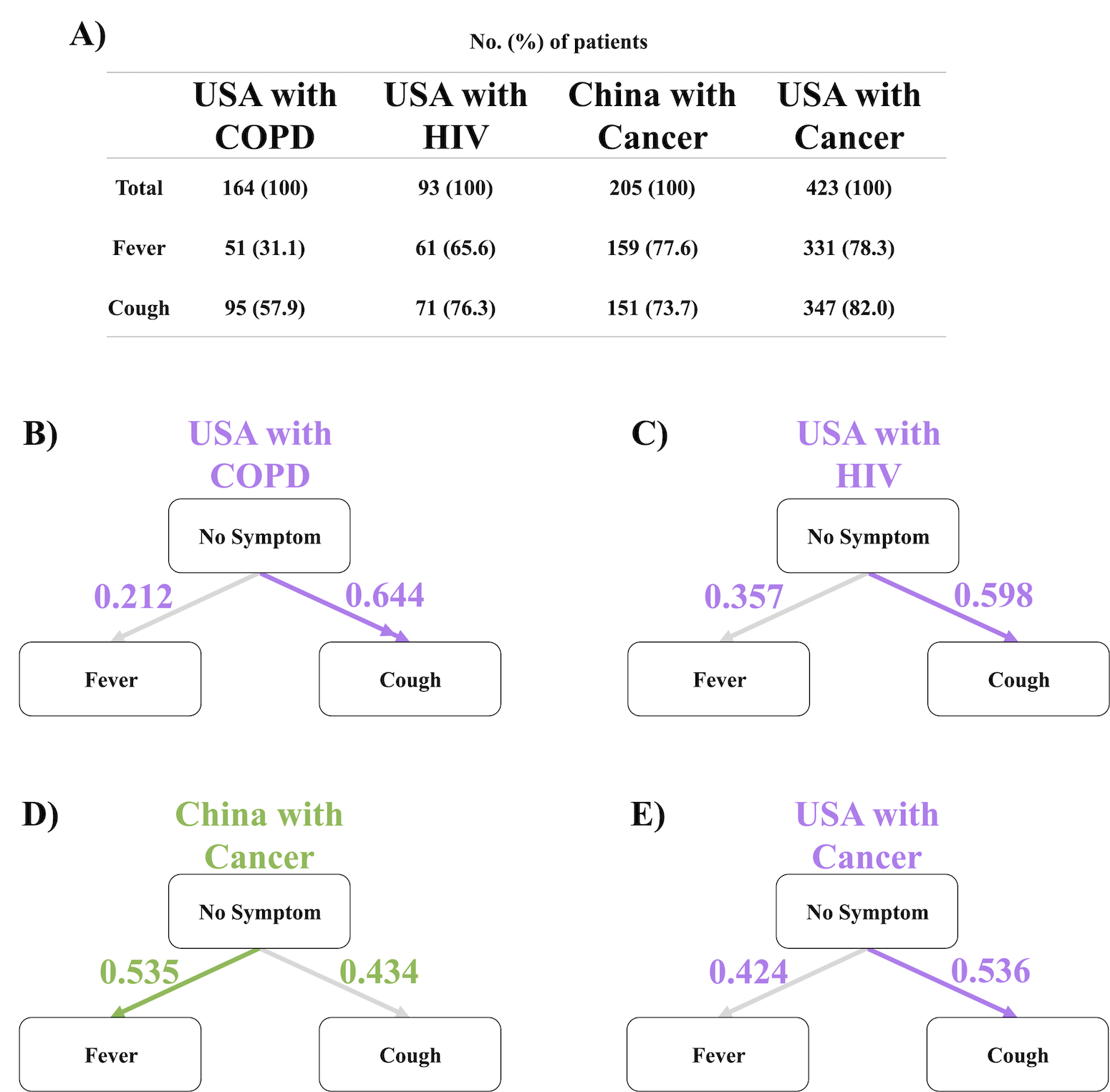

Supplement: S20 Fig — A) Table of raw frequency data specifying the number and the percentage of patients that experienced fever and cough in the datasets including patients with comorbidities, COPD and HIV, in the USA and cancer in China and the USA. B) Abridged Hasse Diagram depicting the transition probabilities from no symptom to fever or cough in patients with COVID-19 and COPD in the USA [35]. The double arrow signifies that the transition probability of the most likely first symptom is more than double the transition probability second most likely first symptom. The error of the transition probabilities is 0.061. C) Abridged Hasse Diagram depicting the transition probabilities from no symptom to fever or cough in cases with COVID-19 and HIV in the USA [34]. The error of the transition probabilities is 0.031. D) Abridged Hasse Diagram depicting the transition probabilities from no symptom to fever or cough in cases with COVID-19 and cancer in China [36]. The error of the transition probabilities is 0.026. E) Abridged Hasse Diagram depicting the transition probabilities from no symptom to fever or cough in cases with COVID-19 and cancer in the USA [37]. The error of the transition probabilities is 0.030. (TIF) [file pcbi.1009629.s025.tif]

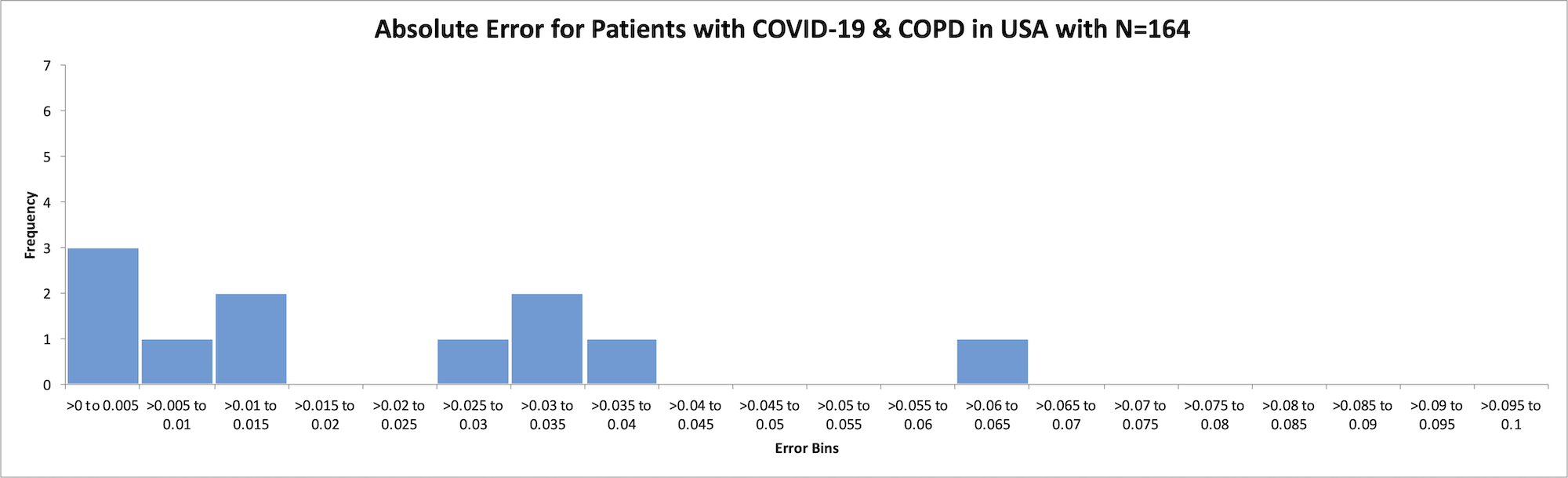

Supplement: S21 Fig — The maximum error was determined from this distribution of absolute error and was used as a conservative measure of error to discern differences in transition probabilities of discernible symptom order. (TIF) [file pcbi.1009629.s026.tif]

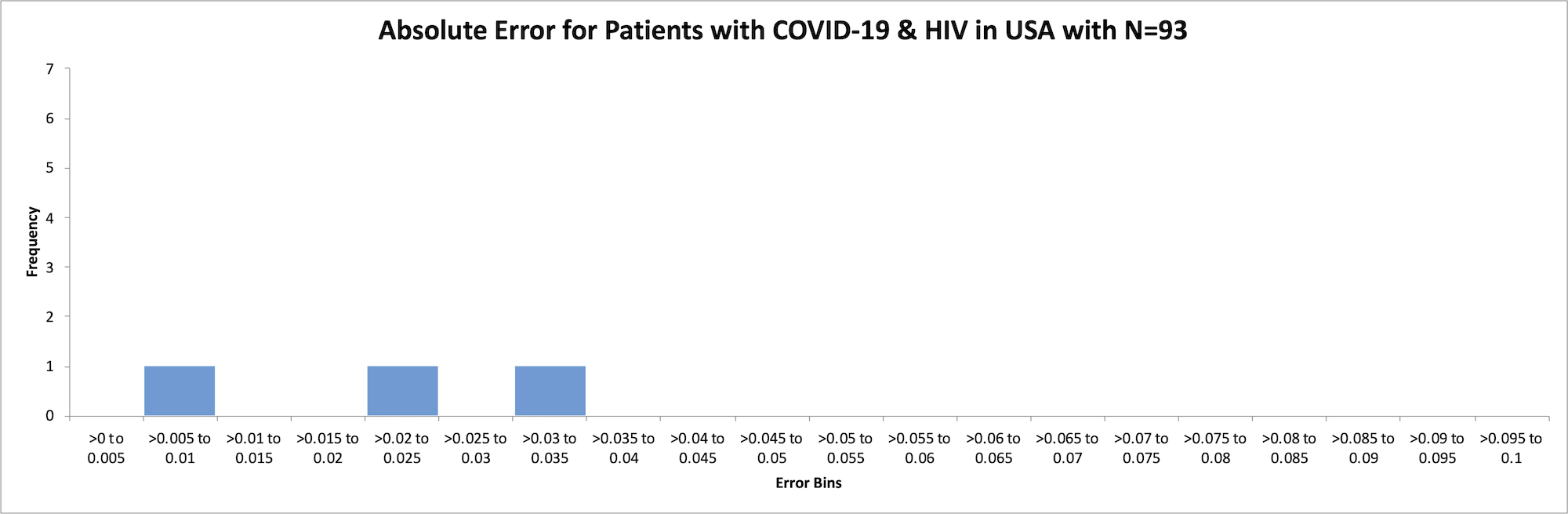

Supplement: S22 Fig — The maximum error was determined from this distribution of absolute error and was used as a conservative measure of error to discern differences in transition probabilities of discernible symptom order. (TIF) [file pcbi.1009629.s027.tif]

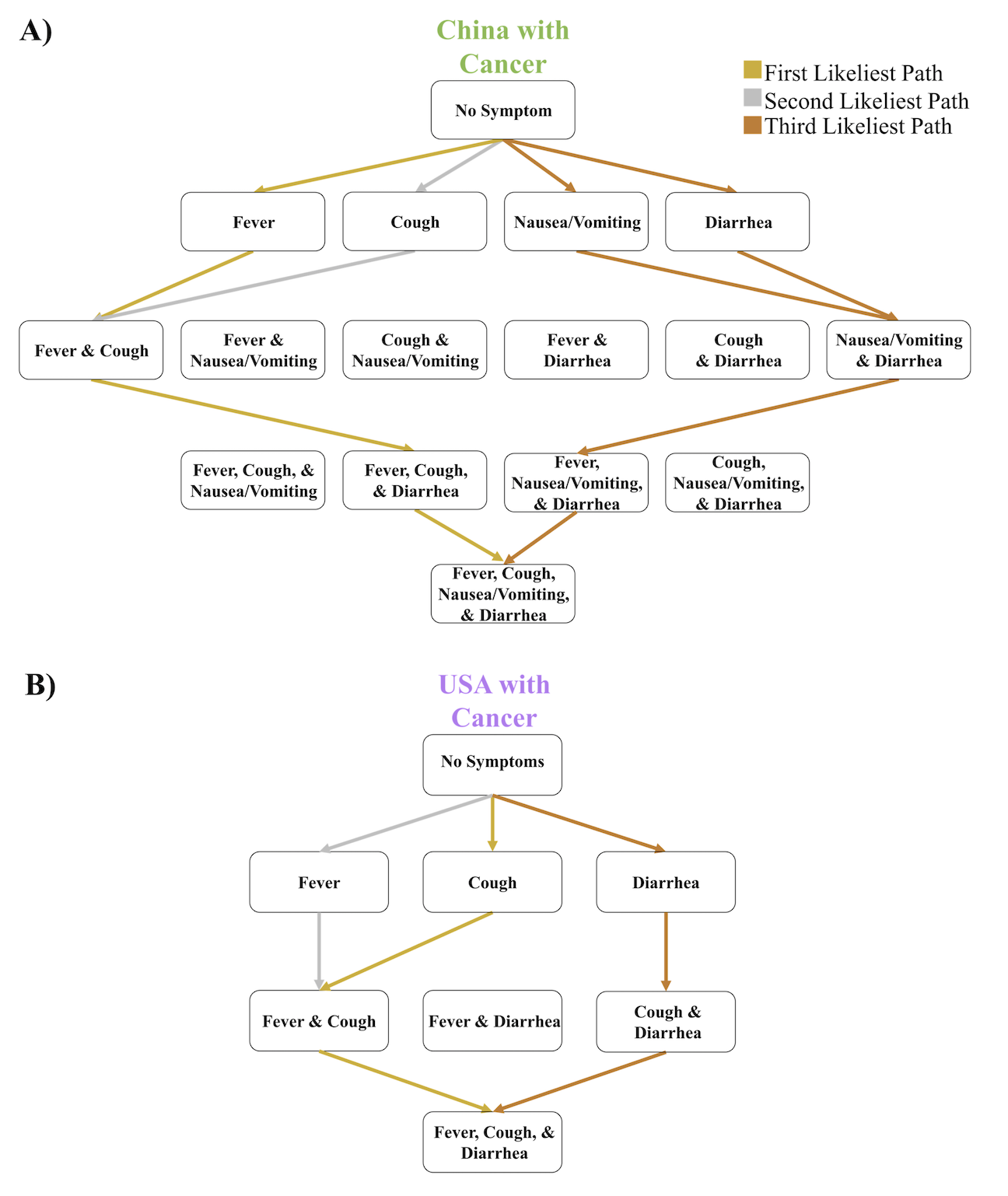

Supplement: S23 Fig — A) The first (gold), second (silver), and third (bronze) likeliest paths representing the order of discernible symptom onset of 205 COVID-19 patients with cancer in Hubei (green). In the case where the gold and silver lines converge, they both follow the same path. B) The first (gold), second (silver), and third (bronze) likeliest paths representing the order of discernible symptom onset of 423 COVID-19 patients with cancer in New York (purple). In the case where the gold and silver lines converge, they both follow the same path. (TIF) [file pcbi.1009629.s028.tif]

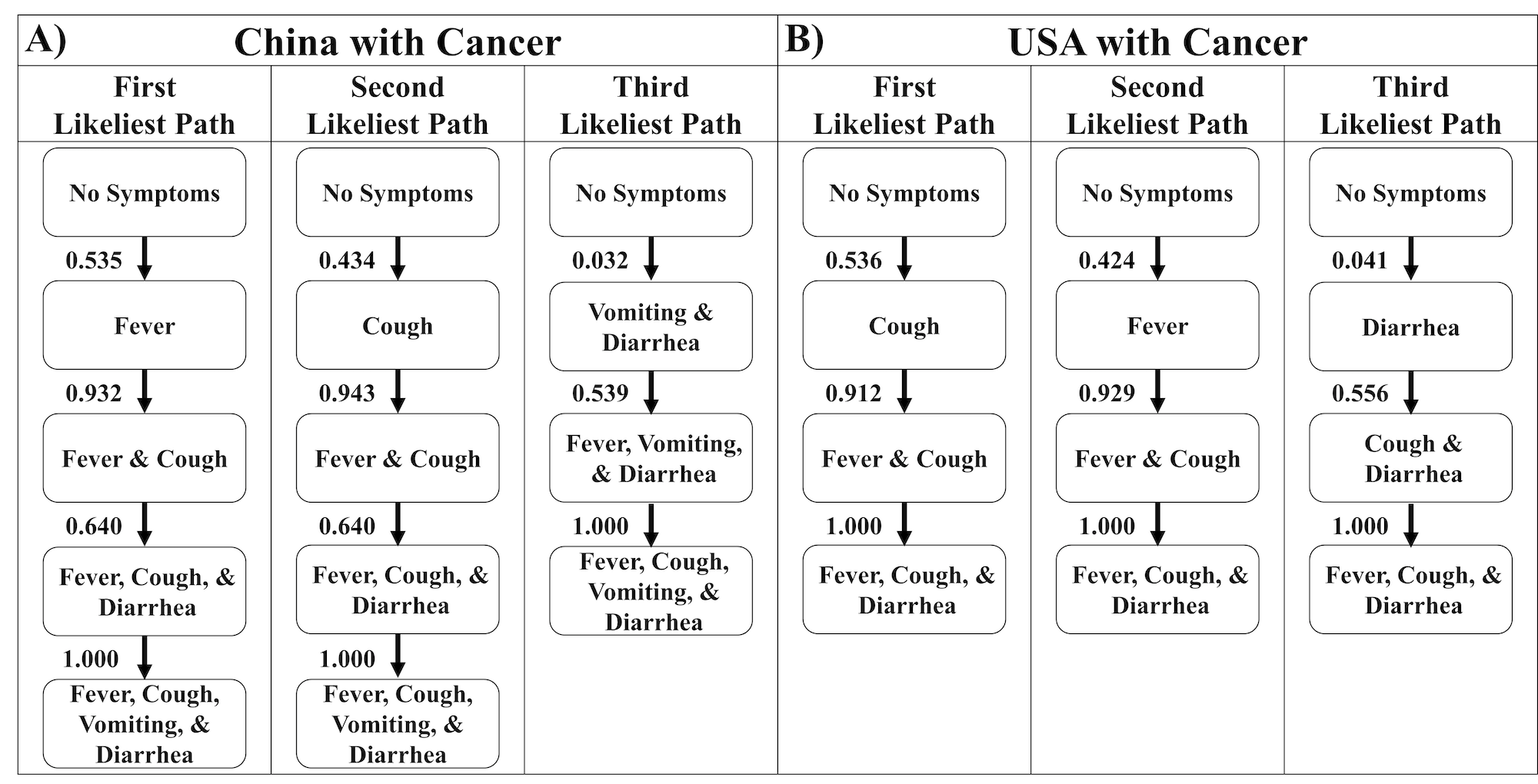

Supplement: S24 Fig — A) The first, second, and third most likely orders of discernible symptoms, with transition probabilities between symptoms of 205 COVID-19 patients with cancer in Hubei. The error of the transition probabilities is 0.026. B) The first, second, and third most likely orders of discernible symptoms, with transition probabilities between symptoms of 423 COVID-19 patients with cancer in New York. The error of the transition probabilities is 0.030. (TIF) [file pcbi.1009629.s029.tif]

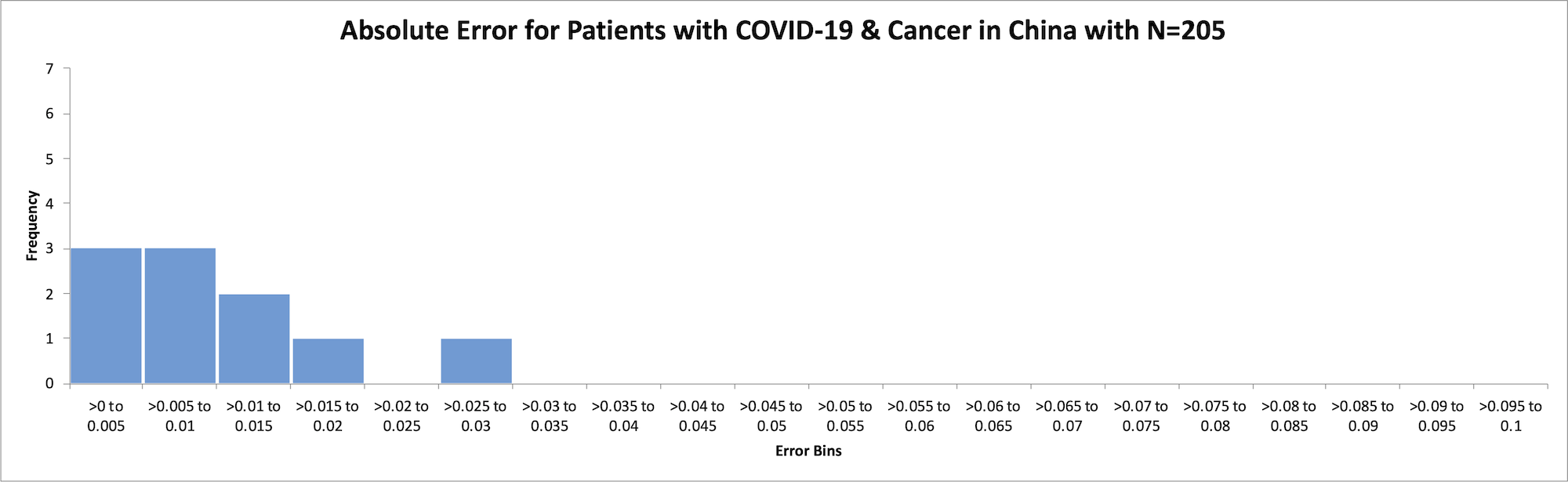

Supplement: S25 Fig — The maximum error was determined from this distribution of absolute error and was used as a conservative measure of error to discern differences in transition probabilities of discernible symptom order. (TIF) [file pcbi.1009629.s030.tif]

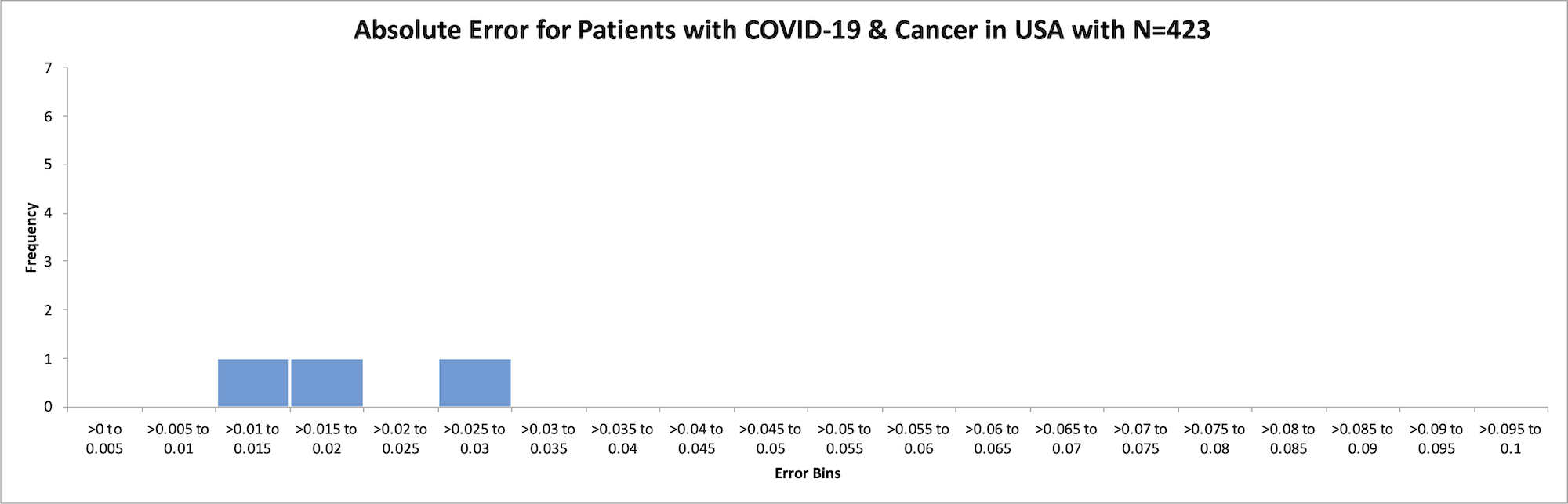

Supplement: S26 Fig — The maximum error was determined from this distribution of absolute error and was used as a conservative measure of error to discern differences in transition probabilities of discernible symptom order. (TIF) [file pcbi.1009629.s031.tif]
