# Supplementary material for: Modeling the onset of symptoms of COVID-19: Effects of SARS-CoV-2 variant
Source: PLoS Comput Biol. 2021 Dec 16;17(12):e1009629. doi: 10.1371/journal.pcbi.1009629 (PMC8675677; doi:10.1371/journal.pcbi.1009629)
Supplement: S1 Appendix — (PDF) [file pcbi.1009629.s032.pdf]

S1 Appendix: Statistics and Demographics of Data

	China[1]	USA[2]	Hong Kong[3]	Brazil[4]	Japan[5] Before D614G	Japan[6] After D614G	Shanghai,[7] China	New York,[8] USA	Atlanta,[9] USA	Comorbidities[10] in China	Comorbidities[11] in USA	Cancer[12] in China	Cancer[13] in USA	COPD[14] in USA	HIV[15] in USA
Sample Size (people)	55,924	373,883	59	67,180	244	2,636	249	393	531	399	463	205	423	164	93
Median Age (years)	51	48	59	59	40 to 49	56	51	62	45 to 64	61 (Mean)	58 (Mean)	63	60 to 69	68 (Mean)	58
Age IQR (year)	36 to 63	33 to 63	44 to 68	44 to 72	30 to 39 to 50 to 59	40 to 71	36 to 64	49 to 74	18 to 44 to 45 to 64	13 (SD)	17 (SD)	56 to 70	50 to 59 to 60 to 69	12 (SD)	52 to 65
Sex (male)	51%	49%	46%	58%	51%	59%	51%	61%	46%	60%	44%	47%	50%	46%	72%
Race	NR	74% not Caucasian	NR	NR	NR	NR	NR	62.6% not Caucasian	21.2% not Caucasian	NR	27.9% not African-American	NR	38% not Caucasian	30.4% not Caucasian	77.4% not Caucasian
Location Data was Collected	23% Not From Hubei, China	All 50 States & D.C.	Hong Kong	SIVEP-Gripe system	Osaka Prefecture of Japan	The COVID-19 Registry Japan	Shanghai	Manhattan Hospital	6 acute care hospitals and associated outpatient clinics in Atlanta, GA	575 hospitals in 31 provinces/ autonomous regions/provincial municipalities across mainland China	Henry Ford Health System in Detroit, Michigan USA	Led by the Wuhan Union Hospital with 9 participating hospitals in Hubei	New York State	Respiratory Institute and the Lerner Research Institute at the Clevelan Clinic in Ohio	Icahn School of Medicine at Mount Sinai in New York
COVID-19 Diagnosis Confirmation	RT-PCR testing of SARS-CoV-2	RT-PCR testing of SARS-CoV-2	Virologically confirmed COVID-19 diagnosis	Molecular diagnostic and clinical epidemiological criteria	Positive PCR test	Poisitve SARS-CoV-2 Test and being in an impatiant care facility	RT-PCR testing of SARS-CoV-2	RT-PCR testing of SARS-CoV-2	RT-PCR testing of SARS-Cov-2	RT-PCR testing of SARS-CoV-2	CDC guidelines interim guidelines for collecting, handling, and testing clinical specimens from persons under investigation (Published February 14, 2020)	RT-PCR testing of SARS-CoV-2 and next-generation sequencing analysis	Detecting SARS-CoV-2 RNA using CDC protocol	RT-PCR testing of SARS-CoV-2	Nucleic Acid Amplification test for SARS-CoV-2
Spotlight Comorbidity Confirmation	NA	NA	NA	NA	NA	NA	NA	NA	NA	NA	NA	Pathologically Diagnosed with a malignant tumor	Diagnosed with Cancer on their electronic medical records (Types of cancer included were leukemia (8%), lymphoma (11%), myeloma (5%), breast (20%), colorectal (9%), lung (8%), prostate (6%), and others (32%), with 56% having metastatic disease)	COPD included on medical chart in HER	HIV on electronic medical record

Note: NR=Not Reported, NA=Non Applicable, IQR=Interquartile Range, and SD=Standard Deviation

References

1. World Health Organization. Report of the who-china joint mission on coronavirus disease 2019 (covid-19). Available on-line: https://wwwwhoint/docs/default-source/coronaviruse/who-china-joint-mission-on-covid-19-final-report. 2020.
2. Stokes EK, Zambrano LD, Anderson KN, Marder EP, Raz KM, El Burai Felix S, et al. Coronavirus Disease 2019 Case Surveillance - United States, January 22-May 30, 2020. MMWR Morb Mortal Wkly Rep. 2020;69(24):759-65. Epub 2020/06/20. doi: 10.15585/mmwr.mm6924e2. PubMed PMID: 32555134; PubMed Central PMCID: PMCPMC7302472
3. Cheung KS, Hung IF, Chan PP, Lung K, Tso E, Liu R, et al. Gastrointestinal manifestations of SARS-CoV-2 infection and virus load in fecal samples from a Hong Kong cohort: systematic review and meta-analysis. Gastroenterology. 2020;159(1):81-95.
4. de Souza WM, Buss LF, da Silva Candido D, Carrera J-P, Li S, Zarebski AE, et al. Epidemiological and clinical characteristics of the COVID-19 epidemic in Brazil. Nature human behaviour. 2020;4(8):856-65.
5. Takeuchi T, Imanaka T, Katayama Y, Kitamura T, Sobue T, Shimazu T. Profile of Patients with Novel Coronavirus Disease 2019 (COVID-19) in Osaka Prefecture, Japan: A Population-Based Descriptive Study. Journal of Clinical Medicine. 2020;9(9):2925.
6. Matsunaga N, Hayakawa K, Terada M, Ohtsu H, Asai Y, Tsuzuki S, et al. Clinical epidemiology of hospitalized patients with COVID-19 in Japan: Report of the COVID-19 REGISTRY JAPAN. Clinical Infectious Diseases. 2020.
7. Chen J, Qi T, Liu L, Ling Y, Qian Z, Li T, et al. Clinical progression of patients with COVID-19 in Shanghai, China. J Infect. 2020;80(5):e1-e6. Epub 2020/03/17. doi: 10.1016/j.jinf.2020.03.004. PubMed PMID: 32171869; PubMed Central PMCID: PMCPMC7102530.
8. Goyal P, Choi JJ, Pinheiro LC, Schenck EJ, Chen R, Jabri A, et al. Clinical Characteristics of Covid-19 in New York City. N Engl J Med. 2020;382(24):2372-4. Epub 2020/04/18. doi: 10.1056/NEJMc2010419. PubMed PMID: 32302078; PubMed Central PMCID: PMCPMC7182018.
9. Killerby ME, Link-Gelles R, Haight SC, Schrodt CA, England L, Gomes DJ, et al. Characteristics associated with hospitalization among patients with COVID-19—Metropolitan Atlanta, Georgia, March–April 2020. Morbidity and mortality weekly report. 2020;69(25):790.
10. Guan W-J, Liang W-h, Zhao Y, Liang H-r, Chen Z-s, Li Y-m, et al. Comorbidity and its impact on 1590 patients with Covid-19 in China: A Nationwide Analysis. European Respiratory Journal. 2020;55(5).
11. Suleyman G, Fadel RA, Malette KM, Hammond C, Abdulla H, Entz A, et al. Clinical characteristics and morbidity associated with coronavirus disease 2019 in a series of patients in metropolitan Detroit. JAMA network open. 2020;3(6):e2012270-e.
12. Yang K, Sheng Y, Huang C, Jin Y, Xiong N, Jiang K, et al. Clinical characteristics, outcomes, and risk factors for mortality in patients with cancer and COVID-19 in Hubei, China: a multicentre, retrospective, cohort study. The Lancet Oncology. 2020.
13. Robilotti EV, Babady NE, Mead PA, Rolling T, Perez-Johnston R, Bernardes M, et al. Determinants of COVID-19 disease severity in patients with cancer. Nature Medicine. 2020;26(8):1218-23.
14. Attaway AA, Zein J, Hatipoğlu US. SARS-CoV-2 infection in the COPD population is associated with increased healthcare utilization: An analysis of Cleveland clinic's COVID-19 registry. EClinicalMedicine. 2020;26:100515.
15. Ho H-E, Peluso MJ, Margus C, Matias Lopes JP, He C, Gaisa MM, et al. Clinical outcomes and immunologic characteristics of Covid-19 in people with HIV. The Journal of infectious diseases. 2020.

	China[1]	USA[2]	Hong Kong[3]	Brazil[4]	Japan[5] Before D614G	Japan[6] After D614G	Shanghai,[7] China	New York,[8] USA	Atlanta,[9] USA	Comorbidities[10] in China	Comorbidities[11] in USA	Cancer[12] in China	Cancer[13] in USA	COPD[14] in USA	HIV[15] in USA
Underlying Conditions/ Comorbidity Information	NR	21.8% with Underlying Condition	NR	83.7% with at least one comorbidity	NR	46.8% with a comorbidity	36.1% had at least one coexisting chronic condition	25.2% had diabetes, 50.1% had hypertension, 5.1% had COPD, 12.5% had Asthma, 13.7% had Coronary Artery Disease, and 136 of 380 are Obese	61% with an underlying condition	67.4% had hypertension, 32.6% had diabetes, 14.8% had cardiovascular disease, 7.5% had cerbrovascular disease, 7% had hepatitis B infection, 6.0% had COPD, 5.3% had chronic kidney disease, 4.5% had malignancy, and 0.8% had immunodeficiency	94% with a comorbidity	52% had a comorbidity besides cancer	100% with cancer, 10% with asthma, 7% with COPD, 20% with diabetes, 20% with cardiac dysfunction, 9% with chronic kidney disease, 51% with hypertension, 45% with systemic chemotherapy, 16% with chronic corticosteroid, 2% with chronic lymphopenia, and 7% with immune checkpoint inhibitors as well as 7% having a major surgery in the last 30 days	100% had COPD, 40.4% had Diabetes, 76.9% had Hypertension, 37.5% had Coronary Artery Disease, 34.4% had Heart Failure, 7.3% had Cancer Currently, 15.3% had Cancer in Remission, 26.5% had Immunosuppressive Therapy, 6.5% had Inflammatory Bowel Disease, 32.9% Smokes a Pack a Year, and 32.2% was a Current Smoker	100% had HIV, 4.3% had Autoimmune Disease, 8.6% had Cancer, 34.4% had Diabetes, 18.3% had Heart Disease/ Coronary Artery Disease/ Congestion Heart Gailure, 52.7% had Hypertension, 26.9% had Lung Disease/Asthma/ COPD, 17.2% had Chronic Kidney Disease, 7.5% had End-Stage Renal Disease, and 5.4% had Solid Organ Transplant
BMI	NR	NR	NR	NR	NR	Median (of 2024 participants) of 23.1 with an IQR of 20.5 to 26	NR	NR	40% are obese (16.9% are NR)	NR	Mean of 33.6 with a Standard Deviation of 8.7	NR	Median between 25.0 to 29.9 with the first quartile between 18.5 to 24.9 and the third quartile between 30.0 to 39.9	Mean of 29.9 with a Standard Deviation of 9.0	Median of 26.7 with an IQR of 23.8 to 29.6
Symptoms Frequencies Reported	Fever, Dry Cough, Fatigue, Sputum Production, Shortness of Breath, Sore Throat, Headache, Myalgia or Arthralgia, Chills, Nausea or Vomiting, Nasal Congestion, Diarrhea, and Hemoptysis	Fever, Cough, Shortness of Breath, Myalgia, Runny Nose, Sore Throat, Headache, Nausea/Vomiting, Abdominal Pain, Diarrhea, and Loss of Smell or Taste	Fever, Cough, Dyspnea, Vomiting, Diarrhea, and Abdominal pain/ Discomfort	Fever, Cough, Dyspnoea, Respiratory Dyscomfort, Oxygen Saturation <95%, Sore Throat, Diarrhoea, and Vomiting	Fever, Cough, Headache, Sore Throat, General Malaise, Mucus/Nasal Obstruction/Sneeze, Low Back Pain, Arthralgia, Diarrhea, Shortness of Breath, and Pneumonia (Most common symptoms)	Fever, Cough, Productive Cough, Bloody Sputum, Fatigue, Shortness of Breath, Diarrhea, Sore Throat, Headache, Dysgeusia, Olfactory Pain, Seizure, and Conjunctivitis (Symptoms Reported on Admission)	Fever, Cough, Fatigue, Dizziness and Headache, Rhinorrhea, Sore Throat, Diarrhea, and Inappetence	Fever, Cough, Diarrhea, Nausea or Vomiting, Myalgias, and Dyspnea (Most common presenting symptoms)	Fever, Cough, Shortness of Breath Headache, Chills, Arthralgia, Myalgia, Sore Throat, Loss of smell, Loss of taste, Gastrointestinal Symptoms	Fever, Conjunctival Congestion, Nasal Congestion, Headache, Dry Cough, Pharyngodynia, Productive Cough, Fatigue, Hemoptysis, Shortness of Breath, Nausea/Vomiting, Diarrhea, Myalgia/ Arthralgia, and Chill	Cough, Nasal Congestion, Dyspnea, Fever, Headache, Myalgia, Anorexia, Nausea, Vomiting, and Diarrhea (Symptoms at Presentation)	Fever, Chill, Cough, Sputum, Chest Congestion, Shortness of Breath, Dyspnea, Nausea or Vomiting, Diarrhea, and Fatigue	Fever, Shortness of Breath, Cough, Diarrhea	Cough, Sputum Production, Dyspnea, Fever, Fatigue, Flu like Symptoms, Loss of Appetite, Diarrhea, and Vomiting	Fever, Altered Mental Status, Congestion, Sore Throat, Cough, Shortness of Breath, Myalgia, Anosmia, Diarrhea, and Headache (Present in Initial Emergency Department Note and Admission Note)
Dates Data Collected	February 16 to 24, 2020	January 22 to May 30, 2020	February 2 to 29, 2020	March 1 to May 31, 2020	February 1 to March 31, 2020	All cases as of July 7th, 2020	January 20 to February 6, 2020	March 3 to 27, 2020	March 1 to April 7, 2020	December 11, 2019 to January 31, 2020	March 9 to 27, 2020	Januray 13 to March 18, 2020	March 10 to April 7, 2020	March 8 to May 13, 2020	March 2 to April 15, 2020

Note: NR=Not Reported, NA=Non Applicable, IQR=Interquartile Range, and SD=Standard Deviation
