# Supplementary material for: Modeling the onset of symptoms of COVID-19: Effects of SARS-CoV-2 variant
Source: PLoS Comput Biol. 2021 Dec 16;17(12):e1009629. doi: 10.1371/journal.pcbi.1009629 (PMC8675677; doi:10.1371/journal.pcbi.1009629)
Supplement: Attachment — Submitted filename: Response to Reviewers.docx [file pcbi.1009629.s033.docx]

*We thank the reviewers for constructive comments. Our responses to the reviewers’ suggestions appear below in italicized blue text and yellow highlighted text reference direct changes in the manuscript.*

Reviewer #1: This paper discusses the order of symptoms after SARS-CoV-2 infections. Using Hasse diagrams and large datasets of self-reported symptoms, comorbidities, and cancer status, the authors study how likely a set of four principal symptoms (fever, cough, nausea/vomiting, and diarrhea) are likely to occur and in which order. They expand on previous work in which they performed the same analysis on early data from Wuhan, before the emergence in the Summer of 2020 of the D614G mutation, which is now the globally-dominant variant. They report that fever and cough interchange as the first most likely symptom in those infected with SARS-CoV-2 without the D614G mutation and those infected with D614G. I found the methodology and analyses sound and the paper to be well-written.

*We appreciate the reviewer describing the main findings of our study and finding the methodology and analyses sound.*

I do have a couple questions:

1) Line 251: The earlier dataset did not report nausea/vomiting, so they weren't included in the analyses of the Japanese data (which is of high interest given that the early data was taken before the emergence of D614G). What is the impact of this on predicted outcomes, since nausea/vomiting is in the third most likely path in the USA dataset, and is also found in the early China dataset? Limitations need be more clearly stated in the discussion.

*We thank Reviewer #1 for raising the important issue that we could not evaluate nausea/vomiting in the Japan datasets. Thus, we cannot claim with certainty that if nausea/vomiting was reported, it would not be the most likely initial symptom. However, our analyses of the Japanese datasets confirm the pattern that fever occurs before cough in the Wuhan reference strain dataset by a high transition probability. In the D614G variant dataset, the initial symptom is either cough or fever. Also, we may confirm that diarrhea as a GI symptom does not interfere with these transition probabilities of fever and cough and that diarrhea was more prevalent in the later Japan dataset than the earlier Japan dataset. These observations are consistent with the most likely paths calculated from other regions characterized by the same strain. However, when comparing the results of China against USA for example, nausea/vomiting occurs third, followed by diarrhea in the most likely path for China, whereas in the most likely path of the USA dataset, diarrhea occurs third, followed by nausea/vomiting. Further analysis is required to confirm that diarrhea becomes an earlier symptom than nausea/vomiting in the D614G variant in comparison to the Wuhan reference strain. So, future work must verify how the variant effects where nausea/vomiting falls in the likely paths. To address this, we modified the Discussion with the following:*

*Lines 470-477: “We confirmed the change in order of fever and cough, but we were unable to compare GI symptoms due to the lack of reporting in the Japanese datasets. It is possible that if nausea/vomiting was reported, it could have been the most likely first or second symptom, but because both fever and cough occur prior to GI symptoms with a high likelihood in all other datasets, it is unlikely. The results of our model presented here suggests that there is an order of symptom onset and this order changes with D614G mutation, but how the variant affects the order of GI symptoms among themselves and relative to respiratory symptoms requires further validation, which requires additional data.”*

2) Lines 269-279: The analysis of Google search terms is a weak point of the present paper, but does not affect the results. Given the prevalence of reporting on COVID-19, it seems impossible to untangle whether increases in specific search terms are related to increased symptoms or increases (global) reporting of symptoms in other countries. Again, limitations should be discussed.

*We thank the review for pointing out this limitation that we had not addressed. Therefore, we further detail these limitations of this data in the Discussion with the following sentences:*

*Lines 442-449: “We also performed an analysis of Google Trends of discernible symptom search terms over early months of 2020, which supported our model’s predicted symptom orders in various countries. Nonetheless, we cannot claim to what extent these trends we observed were related to increased prevalence of COVID-19 in the media and how the coverage varied between countries. As both smartphone applications developed by researchers [35, 36] and clinicians themselves record and report the order of symptoms in COVID-19 patients [37], the conclusions of our model can be validated prospectively with patient data.”*

3) Lines 445-446: Should be updated to reflect the variants of concern now in circulation.

*We agree with the reviewer’s comment. We’ve added the following sentence:*

*Lines 514-519: “Strategies to determine variant without sequencing could be increasingly important during the COVID-19 pandemic as new variants emerge, such as the D614G variant and more recent variants of concern, because they may have increased transmissibility (e.g., the WHO-labeled variants Alpha, Beta, and Gamma), increased virulence (e.g., the WHO-labeled variants Alpha and Gamma), and decreased antigenicity (e.g., the WHO-labeled Beta, Gamma, Epsilon, and Delta) [15, 52-59].”*

Reviewer #2: Larsen et al. here analyzed the symptom data from thousands of SARS-COv2 infected patients in China, US and Japan. They modeled the order of symptom onset, and its potential association with virus mutations.

This is an interesting exercise, and could fit into a modeling journal, albeit the implications are somewhat limited. Clinically, the symptoms and their order are not specific enough to really help in diagnosis. Plus, in the US and Western world in general, there is a widespread access to testing, so I am not sure that this is really needed. This however could be more interesting in countries where access to testing is more limited, but in that case specific data to these countries should be analyzed. The association between symptom order and viral strains is potentially interesting but is very speculative in absence of individual data. Many factors could influence the symptom order (including seasonal change, population characteristics), not to mention the reliability and precision of such self-reported symptom to infer subtle changes in the order of symptom onset. This can nonetheless be an interesting modeling exercise, but much more attention should be given to the data and statistical aspects.

*We are grateful for Reviewer #2’s insightful perspective of our study and noting the high potential interest of the current work. Further, we appreciate the reviewer pointing out not only the limited application of the current work to diagnosis in the USA and Western world because of widespread access to testing and increased vaccination in recent months, but also the potential use of symptom order as a diagnostic tool in countries where access to testing is more limited. We agree, so we have noted in the Discussion:*

*Lines 477-483: “Additionally, recording and studying symptom order also may aid in countries that have limited access to testing as an early indicator of COVID-19. Symptom order data from patients in countries with widespread testing available would indicate whether there is a symptom order of COVID-19 different from other respiratory diseases so that symptom order could be used diagnostically [35, 36]. If a symptom order is verified in countries with widespread testing, data in countries without widespread access to testing would then determine whether the order holds there.”*

*We appreciate the Reviewer’s opinion that association between the symptom order and viral strain is interesting. Accordingly, we integrated the Reviewer’s suggestions to rule out the factors that the Reviewer hypothesizes could affect symptom order [please see responses to specific comments below].*

*Seasonal Change:*

*To investigate any change in order of symptom onset due to season, we compared the most likely order of symptoms in different cities where the same variant was predominant. We considered datasets from Detroit, Atlanta, and New York City occurring during varying dates in regions with very different temperatures. Thus, these three datasets represent different seasons. When implementing the Stochastic Progression Model with these datasets, the resulting most likely orders of symptoms were all consistent and exhibited similar initial symptom transition probabilities to each other and the overall USA dataset. Again, we did the same for datasets in Shanghai, China and Osaka, Japan with varying temperatures, and the most likely paths were consistent with the China dataset characterized by the Wuhan reference strain. We present this analysis in the Results (Lines 247-257), Discussion (Lines 456-458), and Supplemental Figures 4 and 13.*

*Population Characteristics:*

*According to the Reviewer’s suggestion, we next investigated whether population characteristics influence order of symptom onset. Previous studies that reported on the first few months of the pandemic have concluded that specifically age seems to be an indicator of a fatal case of COVID-19 [1, 2], but a more recent study found that age was not an indicator of symptom severity [3]. Indeed, more work is required to determine whether there is an effect of age on symptomatic cases [4]. In our study, the median patient ages in the datasets used to study the variant varied between 48 and 62 years old (see S1 Appendix). Given that one study reported that the risk of death is constant until age 59 and doubles after the age of 60 [2], we studied by age a dataset that had the D614G variant predominant and a dataset that had the Wuhan reference strain predominant.*

*Specifically, we reexamined the earlier dataset from Japan in which the Wuhan reference strain was dominant. Consistent with our other Wuhan reference strain findings, the most likely path of every age group had a high transition probability of fever occurring first. Similarly, the most likely orders of symptoms by age from the USA dataset (featuring the D614G variant) all exhibit a close transition probability of fever and cough occurring first, which is consistent with the orders from other datasets featuring the D614G variant. Thus, the transition probabilities of the initial symptom in each group suggests that there is not an effect of age on likely symptom order that would be affected by the patient characteristics of the datasets. These results are described in the Results (Lines 206-213 and 312-318), Discussion (Lines 456-458), and Supplemental Figures 8 and 9.*

*Reliability and Precision of Self-Reported Symptoms:*

*As the Reviewer states, there are concerns about the reliability and precision of self-reported symptoms, which we investigated in our previous work [5]. We compared our model’s performance using symptoms that can be reliably self-reported and those that cannot. For the former, we implemented the model using fever, cough, vomiting, and diarrhea, which are objective and can be measured or observed. When using these four discernible symptoms, our model resulted in distinct paths. For the latter, we implemented the model using the same datasets, but also included myalgia, headache, and sore throat, which cannot be measured, are interpreted differently by different patients, and are less reliable. Indeed, using our model, the most likely symptom orders of these seven symptoms were less precise. Often transition probabilities were too similar to determine a clear next likely symptom. Thus, the four discernible symptoms, which are less subjective and reported more accurately, lead to more precise model results and are the focus in this paper. In the Introduction, we wrote:*

*Lines 88-92: “In our previous work, we determined that there are distinct most likely orders of discernible symptoms for individuals infected with COVID-19 and influenza, which suggests that it might be possible to differentiate diseases by order of discernible symptoms. Discernible symptoms are objective and easy for patients to recognize and amenable to self-reporting.”*

*Inferring Subtle Changes of Symptom Order:*

*As the Reviewer states, there may be difficulty inferring subtle changes in order of symptom onset. The difference in transition probabilities between two paths indicates whether the order of each symptom is distinguished subtly or not. As a result, we made a distinction in our Figures that when a transition probability is greater than two times that of the next likely symptom, we use the convention of a double arrowhead (Figures 1-3). Consistently, we observe that for the Wuhan reference strain, fever occurring first meets this criterion. In addition, for the D614G variant, cough consistently occurs first but never meets this criterion.*

*Specifically, the transition probabilities indicate that fever is 54.8% more likely than cough to occur first in the dataset from China, whereas cough is more likely to occur first in USA by 12.2%. Similarly, in the dataset from Shanghai, fever is 84.0% more likely than cough to occur first, and cough is more likely to occur before fever by 6.2% in the dataset from New York. In agreement, fever is 60.8% more likely to occur before cough in the Wuhan reference strain dataset from Japan, and cough is 1.6% more likely to occur first in the D614G variant dataset from Japan. These results demonstrate that fever is substantially more likely to occur first with the Wuhan reference strain, but cough is more likely to occur first with the D614G variant. Even if this latter result is considered subtle such that cough and fever occur first at the same rates with the D614G variant, our model’s prediction that fever occurs first with the reference strain while fever and cough occur first at similar rates in a variant does not rely on subtle changes.*

• Clarify where the data can be accessed to, how the data look like without any modeling. This is critical to ensure data transparency and reproducibility. I don’t see any basic statistics to give a sense of the data to the reader, nor a sense on how much the modeling exercise is reasonable in regard to the information available. How many individuals, their basic socio-demographic characteristics (age, BMI), what are the symptoms collected, for how long, the prevalence of each symptom over time since symptom onset, how many are pcr-confirmed, how many are antibody confirmed…

*We are grateful to the reviewer for identifying where the manuscript needed more explanation. The raw data used for our models is in Supplemental Tables 1-4. In the Materials and Methods subsections, “Data Collection to Study Variant” (Lines 521-587) and “Data Collection to Study Comorbidities” (Lines 588-672), and S1 Appendix, we’ve included patient characteristics, such as gender and age, race, comorbidities, and BMI, how the original study confirmed infection of SARS-CoV-2, and all symptoms recorded. Additionally, information about data availability is provided in the online submission system as follows:*

*In addition, aspects of our findings here are consistent with prospective studies of symptom progression, which suggests that the data we included is reasonable. Specifically, we describe in the Discussion:*

*Lines 498-503: “Other researchers have studied symptom progression using prospective data collected in real-time and found that fever and cough occur early after infection with similar likelihood in the USA and United Kingdom [35, 49]. These data are consistent with our findings, as these countries have a predominance of the D614G variant [15]. Similarly, a clinical study performed in China found that the most common first symptom in COVID-19 patients was fever, and this was unaffected by comorbidity [50].”*

• Statistical aspects: very unclear how one path is preferred to the other, what are the statistical criterion associated with the decision inferred? As the paper stands, it is very difficult to understand the methodology, and how the statistical models have been chosen, as well as the confidence associated with each preferred path.

*Thank you for pointing out where our original manuscript was unclear. We chose to model the progression of symptom onset by applying a Markov Process to a Hasse Diagram, which represents a graded poset, because these are well-studied methods that are successful at modeling progression using cross-sectional data, and we previously used this strategy to distinguish COVID-19 and influenza patients by symptom order with acceptable confidence (i.e., recall and selectivity). We detailed this in the Introduction:*

*Lines 81-89: “Using this graphical model called the Stochastic Progression Model, we applied a Markov Process and implemented the model over different datasets that represent symptom frequency in a patient population to find most likely and least likely order of symptoms in these diseases [8]. Similar approaches, that include Hasse Diagrams to represent posets to model progressions as Markov Processes, have been previously used to model the accumulation of mutations in cancer and Human Immunodeficiency Virus (HIV) [9, 10]. Additionally, work in environmental science has included these elements of the theory of posets [11]. In our previous work, we used this model to distinguish individuals infected with COVID-19 and influenza with acceptable recall and selectivity [8].”*

*We rewrote the Materials and Methods subsection, titled “Implementation of the Stochastic Progression Model” (Lines 673-718) to explain in greater detail so we may clarify our methodology. In the Results, we introduce this methodology as follows:*

*Lines 135-144: “Using the previous China dataset and this USA dataset we constructed a Hasse diagram, which illustrates states that represent a set of symptoms experienced and transitions between states that represent the likelihood of moving from one state to another (Figure 1A). Transition probabilities in the model are defined as the likelihood of a patient moving from one state of symptoms to another and are calculated from the raw frequency data through the counting method on a Hasse Diagram we previously used [8]. We use the error of the transition probabilities of each dataset so that we may compare transitions within each implementation and determine the most likely path. Therefore, the edge with the highest transition probability is the most likely initial symptom when that transition probability is greater than the second highest transition probability by an amount greater than the error of the implementation.”*

*We further described in more detail how one path is preferred using the transitional probabilities as the statistical criterion to distinguish the likelihood of paths in each implementation of the model:*

*Lines 706-717: “The error of a specific node was then calculated by a series of steps. First, each path to that node was determined. The transition probabilities of each edge in a path were multiplied to a single product. The product of each path leading to the node together are summed. Finally, that summed value is subtracted from the calculated state probability. The absolute value of that result is the error of that node. The value of the node with the highest error was then determined to be the error of that implementation of the model to serve as a conservative error estimate (Supplemental Figures 1,2,6,7,11,12,14-17,20-22). The error of each implementation allows us to compare each edge within the same implementation. We finally determine which path is more likely by directly comparing transition probabilities of edges. If an edge from a node has a higher transition probability than any other edge from that node by a margin greater than the error, we identify that edge as more likely than any of the others from that node.”*

• The order of symptoms is important but probably even more important is the timing of these symptoms. Can you use the model to project on the timing of onset of these symptoms?

*While we agree with the Reviewer that timing could be even more important, the importance of symptom timing is not yet defined, whereas the association of order of symptoms to characteristics of disease presentation in the patient is well-documented and has been suggested to be critical to study towards disease progression. Specifically, multiple studies have investigated whether GI symptoms indicate specific characteristics of a more severe disease of COVID-19. For example, one study found that patients with abdominal pain were almost four times more likely to experience severe COVID-19 [6]. Another study of COVID-19 patients who experienced mild severity early digestive symptoms found that these patients experienced the disease for a longer duration and were diagnosed later [7]. Both of these studies suggest these patients who experienced an onset of GI symptoms before respiratory symptoms are a subgroup of COVID-19 disease presentation.*

*We hope to study the timing of symptom onset in the future. For example, if we had time-series data where patients report symptoms as they appear daily, we can infer timing along edges of the Hasse Diagrams between two symptoms by finding the average and standard deviation of days between symptom onset for all patients that experience that specific order of symptoms that the edge represents. We address this in the Discussion:*

*Lines 497-498: “Also, the time between symptoms and the time after infection or diagnosis that a symptom occurs could be investigated.”*

*We thank the reviewers again for the constructive feedback. We hope we have satisfied the issues that the reviewers have raised, and that PLoS Computational Biology will consider accepting the manuscript for publication.*

References

1.	Zhou, F., et al., *Clinical course and risk factors for mortality of adult inpatients with COVID-19 in Wuhan, China: a retrospective cohort study.* The lancet, 2020. **395**(10229): p. 1054-1062.

2.	Porcheddu, R., et al., *Similarity in case fatality rates (CFR) of COVID-19/SARS-COV-2 in Italy and China.* The Journal of Infection in Developing Countries, 2020. **14**(02): p. 125-128.

3.	Chang, M.C., et al., *Risk factors for disease progression in COVID-19 patients.* BMC infectious diseases, 2020. **20**(1): p. 1-6.

4.	Omori, R., R. Matsuyama, and Y. Nakata, *The age distribution of mortality from novel coronavirus disease (COVID-19) suggests no large difference of susceptibility by age.* Scientific reports, 2020. **10**(1): p. 1-9.

5.	Larsen, J.R., et al., *Modeling the Onset of Symptoms of COVID-19.* Frontiers in Public Health, 2020. **8**: p. 473.

6.	Henry, B.M., et al., *Gastrointestinal symptoms associated with severity of coronavirus disease 2019 (COVID-19): a pooled analysis.* Internal and emergency medicine, 2020. **15**(5): p. 857-859.

7.	Han, C., et al., *Digestive symptoms in COVID-19 patients with mild disease severity: clinical presentation, stool viral RNA testing, and outcomes.* The American journal of gastroenterology, 2020.
