# Supplementary material for: Modeling the onset of symptoms of COVID-19: Effects of SARS-CoV-2 variant
Source: PLoS Comput Biol. 2021 Dec 16;17(12):e1009629. doi: 10.1371/journal.pcbi.1009629 (PMC8675677; doi:10.1371/journal.pcbi.1009629)
Supplement: Attachment — Submitted filename: Response to Reviewers.docx [file pcbi.1009629.s034.docx]

*We thank the reviewers for constructive comments on the first revision. Our responses to the reviewers’ suggestions appear below in italicized blue text and italicized red text reference direct changes in the manuscript.*

Reviewer #1: The authors have responded to my comments, thank you.

*We are grateful for Reviewer #1’s recognition of the changes made in the first revision.*

In their revisions, the authors note

"To investigate these possible factors, we determined the most likely orders of symptom onset for various datasets from different cities in the USA (Detroit, Michigan, New York, New York, and Atlanta, Georgia)"

and performed similar analyses in both China and Japan. It is unclear to me whether data for these cities were included in the original country-level datasets? If so, these results are not surprising, and the analysis would need to be redone using independent data (validation step).

*We appreciate Reviewer #1’s suggestion here. The patient characteristics of studies conducted at cities were collected during overlapping dates as the patient characteristics of the corresponding country-level studies, and we could not rule out that these datasets are dependent. To this end, we’ve implemented the model on datasets independent of other datasets we use in this work and changed the Results Section titled “Symptom order correlates with variant of SARS-COV-2” and* ***Figure 3*** *to focus that section on validation from independent datasets.*

*Additionally, information about code availability is provided in Materials and Methods as follows:*

*Lines 749-750: “The code can be viewed at* [*https://github.com/j-larsen/Stochastic_Progression_of_COVID-19_Symptoms*](https://github.com/j-larsen/Stochastic_Progression_of_COVID-19_Symptoms)*.”*

Reviewer #2: I thank the authors for their detailed response to my comments. Some of my initial comments have been well addressed, but I still have comments on the data description and statistical aspects for your consideration and that of the Editor.

*We thank Reviewer #2 for acknowledging the improvements to the manuscript and carefully reading the revision.*

• Thank you for adding the raw data. It appears from Table S1 that there is a huge heterogeneity in the data used. Data coming from cities, as well as data in individuals with comorbidities/cancer are very sparse. Since the results are not different than in the general population, I would recommend to put most of these analyses in the supplementary and to focus on the main message.

*We agree with Reviewer #2 to put a greater focus on the main message in the main manuscript. Therefore, we have reduced the discussion of the comorbidities and factors that do not affect order, such as climate and age, to one section of the Results titled “Climate, age, and comorbidities do not affect symptom order,” and we have moved figures focused on comorbidities to the Supplement.*

• Instead I would recommend to put the description of the data in the main manuscript (focusing on the data that are used in the main). Following my initial comment, it is very important that the model predictions do not appear as a black box. Even if PLoS CB is a modeling journal, readers need to know what information is immediately visible from the data, and which one is extracted from the model. I would therefore recommend to have a figure showing, for the main data used, the raw frequency data, focusing on the first and second symptoms. We need to get a sense of the data and not only the results.

*Reviewer #2’s suggestion to include the description of the data in the main text is helpful. To this end, we have added a table that contains the raw frequency data of the first two symptoms to* ***Figures 1, 3,*** *and* ***4,*** *to allow for comparison between the data and the results.*

• Regarding statistical aspects, I would recommend to systematically include confidence interval along with every point estimate of transition probability. This is particularly important given the heterogeneity in the population size used in the study.

*Because we do not have a formal metric representing a confidence interval or confidence associated with each specific path (please see further description in the response to the following comment), we have included a description of our error estimate in the Results section, as well as an explicit statement of error for each dataset in the main text as they appear in the Results section. This error estimate is how we distinguish one likely path from another in each implementation.*

• If I understand correctly lines 706-717, there is no formal statistical test to determine a type I error and how much confidence we have that one path is preferred to another. If there is no possibility to test the likelihood of symptom order (at least the two first one), this should perhaps be added in the discussion

*Reviewer #2 correctly recognizes the limitations to the model. We now address this further in the discussion.*

*Lines 499-508: “Currently, there is no formal metric describing the confidence associated with each specific path of likely symptoms, but in our previous application of the Stochastic Progression Model to likely symptom order of COVID-19, we were able to distinguish COVID-19 and influenza patients by symptom order with acceptable recall and selectivity [9]. Until we develop an appropriate confidence interval for the Stochastic Progression Model, we use a conservative error estimate based on state and transition probabilities to differentiate likely paths within each implementation of the model. As a result, we cannot explicitly state the confidence of each likely path. We are not proposing that the model predicts the likelihood of symptom order. Instead, we note that there are distinct features of symptom progression by datasets that correlate with the predominate viral variant.”*

• The public health implications should be clarified. I am not sure to understand the rationale for “support the practice of reporting the order of symptom onset from patients”. I don’t think that one would expect that this reporting could substitute or even complement viral sequencing. This is not a problem for a publication, the interest of the approach lies in its description of potentially different symptom order across viral strains, and the possibility that it may be one additional factor for increased transmission associated with D614G, but I don’t see the need to “oversell” the approach. Please consider modify these aspects in the abstract and in the discussion.

*We thank Reviewer #2 for helping us better communicate our message. We have removed the quote above from the Abstract, and we have changed the language in the Discussion to more precisely convey the public health implications of our study.*

*Lines 509-510: “This work highlights the need for new forms of data to be collected to develop strategies to study pandemics and symptomatic patient experience.”*

*Lines 519-520: “Along with these studies, our current study underscores the potential value of symptom order towards understanding disease progression, especially in cases including mutating viral strains.”*

*Lines 529-534: “Strategies to discern key differences in variants could be aided by the observation of symptom order. Such strategies will become increasingly important during the ongoing COVID-19 pandemic and future outbreaks of emerging variants of concern, which exhibit increased transmissibility (e.g., the WHO-labeled variants Alpha, Beta, and Gamma), increased virulence (e.g., the WHO-labeled variants Alpha and Gamma), and decreased antigenicity (e.g., the WHO-labeled Beta, Gamma, Epsilon, and Delta) [16, 55-62].”*

*We thank the reviewers again for the constructive feedback. We hope we have satisfied the issues that the reviewers have raised, and that PLoS Computational Biology will consider accepting the manuscript for publication.*
